# Methods for Estimating the Detection and Quantification Limits of Key Substances in Beer Maturation with Electronic Noses

**DOI:** 10.3390/s24113520

**Published:** 2024-05-30

**Authors:** Julia Kruse, Julius Wörner, Jan Schneider, Helene Dörksen, Miriam Pein-Hackelbusch

**Affiliations:** 1 Institute for Life Science Technologies (ILT.NRW), OWL University of Applied Sciences and Arts, 32657 Lemgo, Germanyjulius.woerner@th-owl.de (J.W.); jan.schneider@th-owl.de (J.S.); 2 Institute Industrial IT (inIT), OWL University of Applied Sciences and Arts, 32657 Lemgo, Germany; helene.doerksen@th-owl.de

**Keywords:** multidimensional sensor arrays, MOS sensors, beer fermentation, process control, gas analysis, metal oxide semiconductors, intentional data analysis, chemometrics, PLSR, PCA, first-order calibration

## Abstract

To evaluate the suitability of an analytical instrument, essential figures of merit such as the limit of detection (LOD) and the limit of quantification (LOQ) can be employed. However, as the definitions k nown in the literature are mostly applicable to one signal per sample, estimating the LOD for substances with instruments yielding multidimensional results like electronic noses (eNoses) is still challenging. In this paper, we will compare and present different approaches to estimate the LOD for eNoses by employing commonly used multivariate data analysis and regression techniques, including principal component analysis (PCA), principal component regression (PCR), as well as partial least squares regression (PLSR). These methods could subsequently be used to assess the suitability of eNoses to help control and steer processes where volatiles are key process parameters. As a use case, we determined the LODs for key compounds involved in beer maturation, namely acetaldehyde, diacetyl, dimethyl sulfide, ethyl acetate, isobutanol, and 2-phenylethanol, and discussed the suitability of our eNose for that dertermination process. The results of the methods performed demonstrated differences of up to a factor of eight. For diacetyl, the LOD and the LOQ were sufficiently low to suggest potential for monitoring via eNose.

## 1. Introduction

Analytical instruments play a pivotal role in scientific research and industrial applications, where they are relied upon to deliver results with high accuracy and reliability. In order to compare instrument performances to each other and to instill confidence in users, the results generated by all analytical instruments should encompass essential figures of merit [1]. Among the most crucial figures of merit are the lower limits of application, specifically the method’s detection (LOD) and quantification (LOQ) limits. These parameters hold significant importance, as their definitions encompass the two analytical concepts of sensitivity and precision [2] and therefore impact the suitability of an instrument for potential applications [3]. In our case, such an instrument is an electronic nose (eNose). Particularly because eNoses often operate near said limits, it is advisable to initially determine the lower limits of application for target substances to assess their suitability for the use of an eNose.

Over the past few decades, eNoses have garnered considerable attention from both the scientific community and various industrial sectors. Originally introduced in 1982 by Persaud and Dodd [4], these devices have undergone significant transformations. They have evolved from bulky and power-intensive instruments into portable, cost-effective, and low-power solutions, primarily due to advancements in materials, sensors, and machine learning technologies [5,6]. The broad range of their potential applications stems from their capacity to detect a wide variety of volatile substances and gases swiftly and cost-effectively without destroying the sample. Further, no time-consuming sample preparation is required, which, however, is typical of traditional gas analysis instruments [5,6]. eNoses have consequently found applications across diverse industries, including agriculture [7], security [8], food [9], environmental monitoring [10] and healthcare [11,12,13].

An eNose typically consists of two core elements: a sensor array and a data processing unit, mirroring the rudimentary structure of the human olfaction process [6]. In essence, the underlying principle of an eNose is based on the interaction of gaseous molecules with the sensor array composed of sensors possessing distinct sensitivities, resulting in a substance-dependent response. It thereby creates a volatile imprint that can be ideally recognized through pattern recognition techniques by the software part of the eNose.

The sensor technologies used in arrays for eNoses are diverse. For those readers seeking additional information about the different sensor types, a comprehensive overview and description can be accessed in [14,15]. Metal oxide semiconductors (MOSs), such as those used in this work, are widely favored sensors due to their extensive industrial availability, cost-effectiveness, sensitivity to a broad range of gases and easy electronic measurement properties [10,16,17]. Their sensitive layer is typically composed of SnO_2_, ZnO, TiO_2_, In_2_O_3_, or WO_3_ if applicable doped with catalytically active platinum or palladium [18]. Its resistance, which is influenced by reversible redox reactions dependent on the surrounding air, is utilized as the analytical parameter and typically measured by two contact electrodes [18].

When assessed individually, MOS sensors are not intrinsically specific to one substance [10]. However, they exhibit varying responses to analyte exposure due to different modifications. These can be attributed to the type of metal oxide used, the manufacturing processes (resulting in varying porosity, grain size and layer thickness) or the operating temperature [10,19]. Specificity can be achieved through the combination of multiple MOS sensors into an array [20]. The number of gas sensors used in an eNose array varies between different projects and use cases, but typically ranges from 8 to 32 [21]. Such an array consequently generates multidimensional data that can be subjected to data analysis methods, allowing for potential sample classification or even identification [20]. MOS sensors commonly exhibit non-linear responses to different analyte concentrations [22]. However, at low concentrations, their behavior can be considered to be quasi-linear, justifying the use of linear models for the LOD determinations introduced in Section 2.3 [22].

eNoses are likely to respond to substances beyond the target compounds. Furthermore, eNoses are known to be sensitive to changes in ambient conditions and, depending on the sensor material, are prone to sensor drift [6]. It is therefore worthwhile to investigate whether observed differences can truly be attributed to process-related target substances. To accomplish this, it is advisable to determine their LODs and LOQs individually and to compare those to the concentrations present in the process [23]. Without this initial step, differences in processing stages detected by an eNose could solely arise from interference substances, drift or ambient conditions unrelated to the monitored process and could cause an erroneous discrimination ability. 

However, determining the LOD and LOQ for a compound using eNoses presents a more complex challenge. This complexity arises from the fact that eNoses incorporate a multitude of sensors within the sensor array, and the results obtained from the system involve multiple dimensions for each sample, also known as first-order data. The established methods for LOD and LOQ determination typically pertain to zeroth-order data, making their application to eNoses less straightforward [24]. Furthermore, different approaches to calculate a LOD present in the literature lead to (sometimes greatly) different limits and thus make it difficult to use them for comparison purposes [25,26,27,28].

Historically, eNoses have been primarily employed for qualitative distinguishability, often relying on methods of machine learning, such as the visual distance in Linear Discriminant Analysis or the classification capability of algorithms. While this has been valuable, understanding the detection limits for process-relevant substances for eNoses offers distinct advantages: it ensures that any observed differences are truly attributed to target compounds rather than external interference, and it provides transferable values useful for various applications. To the best of our knowledge, there is currently no paper focusing on LOD calculation for eNoses. Hence, our aim is to consolidate and compare methods that could be used for this purpose, ultimately facilitating the calculation of more and realistic LODs for eNoses. Although there are publications that calculate LODs for eNoses, these calculations are not transparent, which makes a comparison difficult. In most cases, there is also no justification for the chosen method. For this reason, the LOD calculation for eNoses will now be considered systematically and transparently.

As eNoses have previously demonstrated their utility in real-time beer quality assessment, suggesting potential for their integration into production lines [29,30], we have chosen to analyze this case for eNoses’ use. Another study successfully distinguished the stages of the beer-aging process through eNose analysis [31]. Continuous odor monitoring during brewing is expected to assist brewers in better controlling beer quality through eNose technology [32]. To further investigate the use of eNoses in the beer process, we exemplify LOD determinations by analyzing key substances involved in beer maturation, namely the compounds acetaldehyde, diacetyl, dimethyl sulfide, ethyl acetate, isobutanol and 2-phenylethanol [33]. Current offline measurement methods in the beer process only focus on diacetyl concentration and gravity (extract content) and are time-consuming. The lack of real-time information leads to fixed brewing methods or infrequent manual adjustments. Approaches for real-time diacetyl measurement [34,35,36] can reduce process time and costs by up to 25% [35] and monitoring other metabolic indicators could help control fermentation and maturation for desired beer sensory properties. 

With this paper, our goal is to apply common methods for LOD estimation to the multidimensional scenario of an eNose by presenting and comparing approaches for this problem. Defining the application limits for the mentioned compounds and comparing them to concentrations in the beer production process allows us to initially assess the suitability of an eNose for monitoring and controlling this specific process. 

## 2. Background and Theory

### 2.1. Beer Fermentation and Aroma-Active Volatile Compounds

Aroma, a prominent quality trait in foods and beverages, not only serves as one of consumers’ initial evaluations before tasting a product, but also acts as an indicator of potential quality issues arising from storage conditions, processing contamination, or raw materials [29]. As consumer expectations for beer continue to elevate, there is a pressing need for swift and cost-effective methods to evaluate its aroma quality both during and after production [29]. 

The central bioconversion step in beer production is fermentation. In the primary fermentation, yeast cells convert glucose, fructose, maltose and maltotriose, in the given order, into biomass, different alcohols, carbon dioxide and different flavor components [37]. The secondary fermentation begins after most of the yeast has been cropped and consists primarily of a stabilization and physicochemical maturation process as well as the accumulation of carbon dioxide [33].

**Figure 1:**
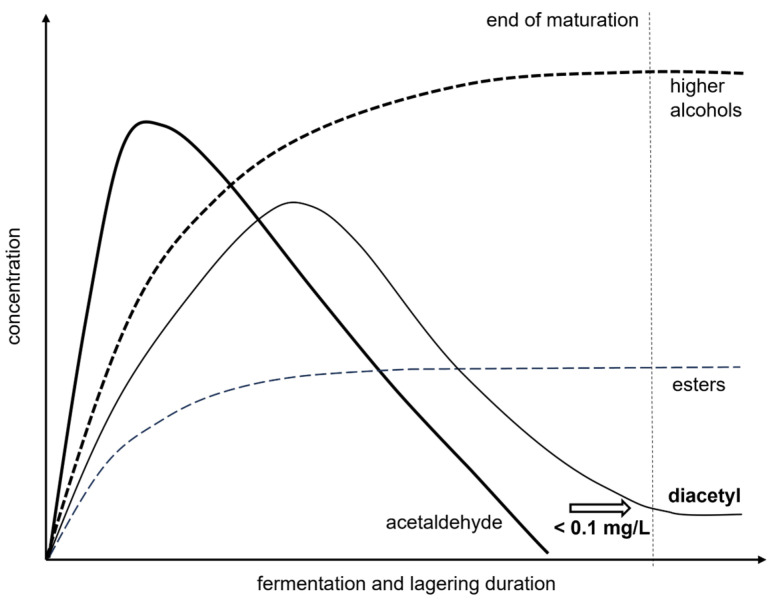
A schematic illustration of the formation and breakdown of fermentation by-products in the beer-making process. The figure is adapted from [48].

**Figure 2:**
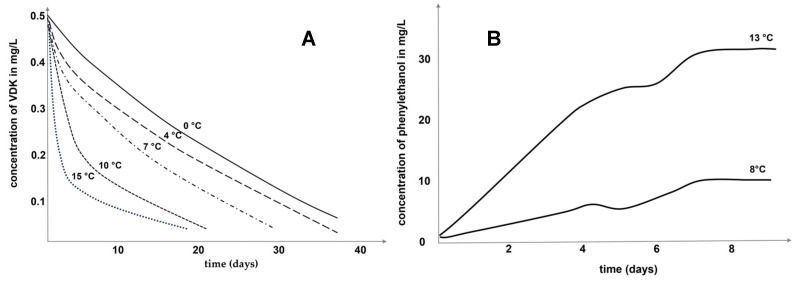
The concentration development of total vicinal diketones (VDK) (**A**) [49] and 2-Phenylethanol (**B**) [50] during the beer fermentation process depending on the temperature and time.

Apart from ethanol and carbon dioxide, fermenting yeast cells also produce a wide range of secondary metabolites. Although they are present in notably lower concentrations, these secondary byproducts play a crucial role in shaping the intricate aroma of the beer, either as an off-flavor or as an enhancer of the overall aromatic profile. An overview of their concentration evolution during the fermentation process can be found in Figure 1. As illustrated in Figure 2 for 2-Phenylethanol and vicinal diketones (VDKs) exemplarily, temperature and time strongly influence their concentrations during fermentation. Therefore, in situ information about the current concentrations of key compounds from the group of higher alcohols, esters, carbonyls and VDKs could be used for targeted temperature adjustment as well as process duration and, thus, to control the process. In the following paragraphs, the role of the substances analyzed in this study within the beer-brewing process will be described. 

Diacetyl (2,3-butanedione) is particularly important among the side-products. It is an aroma-active VDK that is considered an off-flavor compound with a ‘butterscotch’ aroma. Maintaining a low total diacetyl concentration, ideally below 0.1–0.2 ppm in light lager beers, is crucial [38,39]. The brewing process includes a phase called the diacetyl rest as a first part of the secondary fermentation, where the temperature is raised to expedite the reduction in diacetyl performed by the yeast. If the targeted maximum concentration is not reached, the subsequent phase, termed ‘lagering’ (Figure 1), marks the final segment of the secondary fermentation process with lowered temperatures. Furthermore, the presence of VDKs can indicate potential microbial contamination, such as that from *Lactobacillus* spp. or *Pediococcus* spp. [38,40]. The diacetyl concentration is therefore an important quality control parameter in the brewing process. 

Higher alcohols, defined as compounds with longer carbon chains than ethanol, can have both positive and negative impacts on the aroma of beer. Isobutanol (2-methyl propanol), for instance, can have a negative effect on beer quality if its concentration of all higher aliphatic alcohols exceeds 20% [41]. 2-Phenylethanol, on the other hand, is considered to have a positive influence on the aroma of many beers. It is an aromatic alcohol with an intense ‘rose-like’ smell that also burns in its pure form [42]. However, as with aliphatic alcohols, too high concentrations of it can lead to a strong, pungent smell [43]. Phenylethanol is also considered a heat indicator because fast and warm fermentation increases its concentration [42]. 

Esters are formed in relatively large quantities during beer fermentation. Comparably to the higher alcohols, they can have a positive effect on the aroma, but can also lead to an excessively fruity character in too high concentrations [44]. When different esters are present, synergistic effects can arise, influencing the beer aroma even below the individual threshold values [43]. Ethyl acetate comprises the largest amount within the group of esters.

Carbonyl compounds are present in relatively small concentrations in beer [45]. Acetaldehyde is the most important compound, which arises in the metabolic pathway of alcoholic fermentation before its reduction to ethanol. It brings with it a ‘crisp green apple flavor’ [43]. In the matrix of beer, however, a ‘grassy’ off-flavor can occur when the flavor threshold is exceeded.

Sulfur compounds in beer have their origin in the raw materials, yeast metabolism or in microbial contamination. Dimethyl sulfide (DMS), one of the most important off-flavor compounds in beer, originates from the barley. During the germination process in the malting plant, the amino acid methionine is converted into *S*-methyl methionine, the DMS precursor (DMS-P). At temperatures above 70 °C, the DMS-P is partially dissociated to DMS and isothreonine [46]. During the wort-boiling process in the brewery, the total DMS (including its precursors) should be reduced to below 100 ppb because it causes an off-flavor and can make the beer unpalatable [47]. DMS is therefore also an important quality indicator.

To gauge the quantities of these substances, one can make use of their human odor threshold value (OTV), also known as the odor recognition (or detection) threshold. The OTV is the lowest concentration at which the flavor is correctly identified [51] and should not be confused with the stimulus threshold, the lowest concentration at which 50% of a panel perceive a change to a neutral sample. The OTV can be determined in water, in air or in a food. Table 1 summarizes the OTV for the compounds in lager beer that are examined in this paper and are broadly accepted in the brewing science community.

### 2.2. Limit of Detection

The increasing demand for ultrasensitive sensors has resulted in sensors often operating at or near their LOD [56]. Many renowned chemical organizations and unions like the IUPAC, the ICH, the ACS and the ISO have defined the LOD [57,58,59,60]. In summary, this figure of merit is defined as the smallest quantity of a substance that can be detected with reasonable certainty and can therefore be reliably distinguished from a blank. The quantity of a substance can either be its mass or its concentration, depending on the analytical instrument [61]. For eNoses that typically analyze the gaseous headspace of a liquid sample, the quantity refers to the concentration of the analyte in the liquid, as it is known via preparation and as there is a concentration-dependent equilibrium between the sample and the gas space [62].

**Figure 3:**
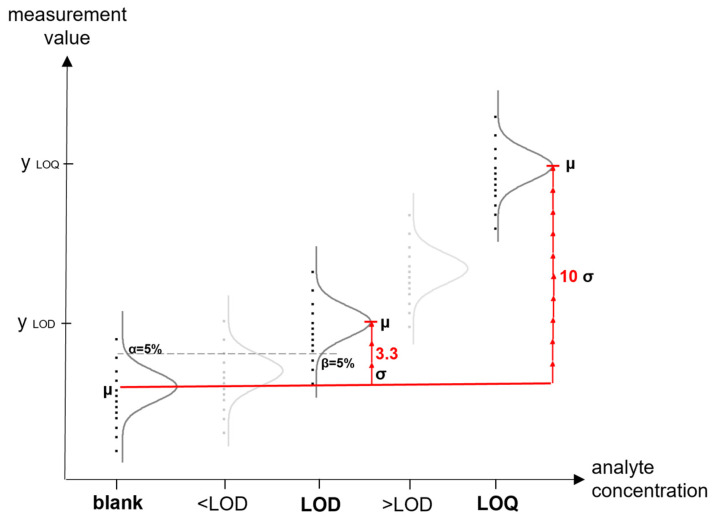
A diagram illustrating the determination of the LOD and the LOQ in accordance with the IUPAC’s definition, utilizing blank determination. Normal distribution and homoscedasticity are assumed.

Various approaches have been established for one-dimensional LOD calculations to determine whether there is a significant difference between a sample and a blank. The following methods are among the most widely adopted. The first method, illustrated in Figure 3, calculates the sensor value exceeding the threshold for achieving the LOD ($y_{LOD}$) by adding a predefined multiple, $k_{LOD}$, of the measured standard deviation of an analyte-free (blank) measurement $s_{b}$ (as an estimate of the true standard deviation σ_b_) to its measured mean value ${\bar{x}}_{b}$ (as an estimate of μ_b_), following Equation (1) and Equation (2) [27,63]:

(1) $$y_{LOD}={\bar{x}}_{b}+k_{LOD}*s_{b}$$

(2) $$s_{b}=\sqrt{\frac{\sum_{i=1}^{N}{{(y_{i}-\bar{y}}_{N_{b}})}^{2}}{N_{b}-1}}$$

$y_{i\;}$ represents the sensor value of blank sample i, ${\bar{y}}_{N}$ denotes the mean of all blank sensor values and $N_{b}$ signifies the number of blank samples.

By selecting different values for $k_{LOD}$, distinct error probabilities for both alpha errors (i.e., false alarms) and beta errors (i.e., misses) evolve. The alpha error concerns the chance of misidentifying a blank as an analyte, while the beta error governs the likelihood of misclassifying a genuine analyte as a blank. This statistical assessment of the LOD was initially introduced by Currie [63] and is accepted by the IUPAC [57]. The IUPAC recommends error probabilities of 0.05 for both alpha and beta errors [57]. These probabilities result in $k_{LOD}$ = 3.3 when assuming homoscedasticity and a normal distribution of both blank and sample values. In the literature, this factor is often simplified to $k_{LOD}$ = 3, which results in higher error probabilities of 0.07 for both errors [64]. For low blank replicate numbers, it is recommended to use Student’s t-distribution and employ $2*t_{\left(1-\alpha ,\;v\right)}$ instead of the general 3.3 for $k_{LOD}$ [57], whereas v corresponds to the degrees of freedom.

The analyte-free measurement, which the method is based on, can either be obtained from the ‘pre-analyte exposure noise’ or by analyzing a blank sample with a 0% analyte concentration, as in [65]. The choice depends on the analytical method applied [66]. Equation (1) solely allows for determining a signal value that must be exceeded by a sample concentration to be considered as the LOD. Consequently, this method can only determine concentrations as LODs that have been measured. 

To determine concentrations as detection limits that have not been directly measured, it is necessary to make use of the calibration curve [58,67]. The curve allows for the determination of an LOD from the measurement value $y_{LOD}$: by leveraging the linear relationship between the concentration and the corresponding signal, the following Equation (3) can be derived from Equation (1) [68]:

(3) $$c_{LOD}=\frac{k_{LOD}*s_{b}}{m}$$

with m being the slope of the calibration curve, called the analytical sensitivity. Instead of using the standard deviation of the blank samples themselves, curve deviations can be used as estimators [67]. For instance, the residual standard deviation (RSD) (i.e., the root mean square error (RMSE)) of the curve, as defined in Equation (4), can be used and interchanged with the $s_{b}$ of the previous method [64,66]. If the calibration samples are sufficiently representative of the test samples, the residuals are comparable to the instrumental noise [69]. Kang et al. discovered that for individual eNose sensors, the residual differences remain consistent throughout the measured range, thus suggesting that the RSD serves as a reliable estimate for noise in determining the LOD [23].

(4) $$s_{residual}=\sqrt{\frac{\sum_{i=1}^{n}{\left(y_{i}-{\hat{y}}_{i}\right)}^{2}}{N-2}}$$

Here, in Equation (4), N is the total number of measurements, $y_{i}$ signifies the observed value for sample i and ${\hat{y}}_{i}$ is the predicted value for sample i. Besides the RSD, the standard deviation of the y-intercept and the standard deviation of the slope have been used as curve deviations to estimate the LOD [64].

Moreover, for all methods making use of a calibration curve, differences in regression techniques can also result in different LOD values [70]. Several methods exist for computing a linear calibration curve, with commonly utilized methods being WLS and OLS regressions. The primary disparity between ordinary least squares (OLS) regression and weighted least squares (WLS) regression lies in handling the variances of the calibration data [70]. Frequently, data exhibits increased variance at higher concentrations, and WLS regression effectively addresses these heteroscedastic errors by assigning greater weight to less variable, more reliable data points. In these instances, WLS regression is recommended for determining the LOD and LOQ [71]. Conversely, OLS regression incorporates all data points equally into the creation of the calibration curve.

In addition to choosing the correct regression technique, various requirements have been outlined for the calibration curve’s calculation. The calibration curve must comprise of samples with concentrations near the LOD and should overlap with the determined quantity [67]. It is recommended that the highest concentration used to determine the LOD should not be significantly higher than 10 to a maximum of 30 times the LOD [61,67]. It is important for the regression procedure that each concentration consists of the same number of replicative signal measurements [67]. Furthermore, the signals from blank samples should generally be incorporated [67]. To account for additional uncertainties associated with the calibration curve based on the chosen concentration levels, the so-called leverage can be incorporated as a factor into the original LOD equation. The magnitude of the leverage provides information about the fluctuations of the intercept and slope, which, in turn, influence the LOD. The higher the leverage, the higher the LOD. Consequently, for example, numerous widely dispersed concentrations will exhibit a lower leverage than fewer closely spaced concentrations. The following Equation (5) incorporating the effective leverage $\frac{1}{n}+\frac{{\bar{c}}^{2}}{\sum {{(c}_{i}-\bar{c}}^{2})}$, which was proposed by Olivieri [72], emerged for the LOD:

(5) $$c_{LOD}=\frac{k_{LOD}*\;s_{residual}*\sqrt{\left(1+\frac{1}{n}+\frac{{\bar{c}}^{2}}{\sum {{(c}_{i}-\bar{c}}^{2})}\right)}}{m}$$

where the $c_{i}$ are the measured concentrations, $\bar{c}$ is the mean of these concentrations and n is the number of samples. An overview of all common equations for LOD estimation is given in Table 2.

### 2.3. Application Limits for Multidimensional Signals

The above-described methods to estimate the LOD for substances in analytical instruments are not directly applicable to eNoses due to the first-order nature of their sensor array’s signal. Therefore, it is not surprising that several findings in the literature imply that LODs for substances measured with eNoses as a whole have not yet been established. Just recently, a review on algorithm designs for eNoses mentioned LOD determination but did not address the challenges of multidimensionality, instead describing the LOD for a single sensor signal [73]. Several studies [65,74,75,76,77,78] that calculated LODs in the context of eNoses calculated the LOD of the substance for individual sensors and differed significantly in calculation formulas (i.e., the signal-to-noise ratio of one vs. ${3.3*s}_{blank}/m$). Just two of those studies reported the LOD taking into account the whole eNose; Feng et al. [65] did so by solely using the most sensitive sensor of the array, and Yang et al. [78] did so by adding the three maximum sensor responses of the array before calculating the LOD.

However, it is essential to utilize data from all sensors in the array because individual sensors lack the necessary specificity to accurately describe the complex characteristics of real-world scenarios and would therefore not be employed individually [79]. As mentioned by Vlasov et al. [80], for multisensor array systems’ ‘electronic tongues’, the detection limits of a sensor array depend not only on the sensing materials but also on the composition of the sensor array itself and can only be determined after data processing, including multivariate data analysis, is completed. Therefore, calculating the LOD when using eNoses should involve integrating data from all sensors intended for real-world use to obtain realistic values. This would ensure an accurate assessment of the eNose’s overall capability for substance detection. 

As stated in the IUPAC technical report from 2006 [69], several authors therefore suggest performing a standard univariate regression using a surrogate signal variable obtained from the multivariate signal. This can then be directly related to the concentration of the analyte [69]. A popular tool to reduce the dimensionality to a singular surrogate signal is principal component analysis (PCA). This is an especially valuable mathematical method for high-dimensional datasets where the features correlate [64] and the number of features greatly surpasses the number of samples, making data exploration and visualization challenging. PCA aims to filter out noise and reveal hidden structures by generating new, uncorrelated components through linear combinations of original features that maximize explained variance [81]. If the explained variance of the first principal component (PC1) is sufficiently high, the value of PC1 could thus become the univariate signal and the usual univariate methods for calculating the LOD would become applicable [82,83]. 

Another surrogate variable for this use could be the concentrations predicted by multivariate regression techniques like partial least squares regression (PLSR) or principal component regression (PCR) [82]. PLSR models the linear relationship between predictor variables and response variables by seeking orthogonal X-scores to predict Y while modeling X, minimizing residual differences with a minimal number of latent variables [84]. PCR, on the other hand, uses principal components as predictors that each maximize the explained variance to reduce multicollinearity and improve regression performance. As Ortiz et al. [85] have proven mathematically, the LOD is invariant for linear transformations of the response variable. The predicted vs. the measured concentration graph can subsequently be used for typical univariate LOD estimation. 

### 2.4. Limit of Quantification

The introduction of the LOQ became necessary due to the limitations of the LOD for quantitative analysis [67]. The LOQ, defined as the minimum concentration or mass that a given procedure can reliably use for quantitative analysis, serves as the threshold below which the analytical method cannot deliver results with acceptable precision [63,66]. In line with Currie’s approach on the LOD, the LOQ involves the addition of a multiple of the standard deviation to the mean of the blank measurements [63]. However, in this case, the standard deviation pertains to the LOQ concentration, not the blank [63]. Assuming homoscedasticity, the deviation can be exchanged [66]. Currie suggests the reciprocal of the acceptable precision as the factor k [63]. For example, if the author deems a 10% relative standard deviation acceptable, the factor k used is 10 [63]. This factor is also recommended by other sources like the ACS and the ICH [58,86]. Assuming homoscedasticity and a normal distribution, the same above-mentioned methods can be applied for LOQ determination as those for the LOD, with the only difference being the replacement of factor k with 10 [87]. The calibration curve requirements remain applicable, which means that the range for determining the LOQ should be adjusted accordingly. Figure 3 illustrates this difference in the value for $k_{LOD}$.

## 3. Materials and Methods

### 3.1. Experimental

#### 3.1.1. Sample Preparation

In this study, six different substances (Isobutanol: for synthesis, Merck KGaA, Darmstadt, Germany; Ethyl acetate: ≥99.5% (ACS), Merck KGaA, Darmstadt, Germany; 1.09623.2500; Diacetyl: 99%, Thermo Scientific Chemicals, Waltham, MA, USA, A14217; Dimethyl sulfide: ≥99%, Thermo Scientific Chemicals, Waltham, MA, USA, 022949.AK; Acetaldehyde: for synthesis, Riedel-de Haën AG, Seelze, Germany; 2-Phenylethanol: ≥98%, Thermo Scientific Chemicals, Waltham, MA, USA, A15241.30) were chosen for eNose analysis. A dilution series was prepared for each substance on the day of measurement. The series were prepared using an LLG Labware pipette (100–1000 μL single channel pipette, Lab Logistics Group GmbH, Meckenheim, Germany). The diluent employed in this study was a 5% *v*/*v* ethanol solution made with distilled water and ethanol (99.5%, VWR Chemicals, Radnor, PA, USA, 85033.320). The selection of the diluent was driven by the objective to better simulate the composition of beer, considering the well-known fact that ethanol in beverages can, in part, mask the sensor array’s response to other volatile compounds [88]. The concentrations were chosen to include values both below and above the estimated LOD, while avoiding excessively high concentrations.

#### 3.1.2. Measurement

The eNose used (Smelldect GmbH, Deckenpfronn, Germany) features 62 SnO_2_ nanowires, which are excited using ultraviolet light. Compressed air is the carrier gas at a flow rate of 100 sccm/min. To maintain stable humidity during measurement, the air passes through a gas washing bottle, setting the relative humidity at 100%, which corresponds to approximately 70% in the sensor chamber due to temperature differences. The sample is placed in a sample vial containing a total volume of 250 mL. For each measurement, 50 mL of the sample was deposited into this vessel. The complete setup can be found in Figures S1 and S2 in Supplementary Materials.

Each measurement cycle of the system consists of three phases (A, B and C). Initially, in Phase *B*, both the sensor and the sample bottle undergo flushing to recover the sensor’s surface and replace the ambient air present in the sample bottle via the compressed air. Subsequently, in Phase A, air is exclusively flushed over the sensor to facilitate the establishment of a liquid–gas equilibrium above the sample. A baseline is thus recorded in the two previously mentioned phases. Finally, in Phase C, the actual sample measurement of the headspace of the sample is conducted. To minimize residual substance effects despite flushing, samples were measured in ascending concentrations. Finally, the sensor array is flushed again via Phase *A* to clean the sensors. To ensure an adequate dataset and maintain realistic variance, each substance was measured on a minimum of three different days. The entire dilution series of one substance was measured per day, and each concentration was measured thrice. This resulted in a dataset of a minimum of 45 data points per substance.

#### 3.1.3. Data Preprocessing

To control the eNose and generate a .csv file for each day, the software provided by Smelldect (Kamina Observer Version 2.0, Karlsruher Institut für Technologie (KIT), Karlsruhe, Germany, 2013-202) was used. The .csv file contains the sensor resistance values for the measured samples of a day. From these time series, individual sample measurements were separated, resulting in a .csv file being created for each individual sample. These files include the resistance curves of all sensors over the measurement phases. Features describing the sensor response can then be extracted from the sensor signals of the eNose. We chose to use the feature $R_{i,d}$ described by Equation (6):

(6) $$R_{i,d}=\left({\bar{y}}_{sample,i}-{\bar{y}}_{base,i}\right)-\frac{1}{n_{d}}\sum_{j=1}^{n_{d}}\left({\bar{y}}_{ethanol,j}-{\bar{y}}_{base,j}\right)$$

where ${\bar{y}}_{sample,i}$ is the mean of the last 10 resistance values during the sample measurement of the i-th sample and ${\bar{y}}_{base,i}$ corresponds to the mean of the last 10 baseline resistance values before the i-th sample measurement. The variable $n_{d}$ denotes the number of ethanol samples on day d. By subtracting the baseline signal before each measurement, a simple baseline correction is performed. By additionally subtracting the signal of the analyte-free ethanolic solution, the influence of the drift between different days is reduced. As a consequence of the feature extraction process, 62 values were obtained for each measurement and subsequently used for LOD determination. Therefore, second-order data (in a matrix) is transformed into first-order data (in a vector). The features were then normalized using z-score normalization.

#### 3.1.4. Calculating the LOD and the LOQ

We utilized data from three different days for each substance to obtain realistic values for the LOD and the LOQ, as eNoses often suffer from poor reproducibility due to sensor drifts [89]. To roughly assess the trends, homoscedasticity, and linearity, and potentially exclude higher concentrations, we relied on residual plots. Given the nature of our data, we considered a residual plot sufficient, and precise adherence to the 0.05 error probabilities is less critical for our purposes of evaluating device suitability. 

The methods outlined in Section 2 were implemented using Python 3.9.13, including PCA, PCR, and PLSR. For $k_{LOD}$, Students t-distribution was utilized, which resulted in a factor of 3.72 for 9 blank samples. The first approach, denoted as PCA I in the following, calculates the LOD as the lowest concentration whose mean value of the first principal component exceeds $y_{LOD}$, which is calculated using Equation (1). For the other method employing PCA (PCA II), a regression line was fitted using OLS to the first principal component against the concentration. The slope and the RMSE were then calculated from this line to estimate the LOD using Equation (5). This process was conducted once using the RMSE and once using the $s_{blank}$ as estimates for $\sigma_{b}$.

To ascertain the optimal number of latent variables included in the PLSR and the PCR, 5-fold cross-validation was used. In this way, a number of components could be selected that do not lead to overfitting and enable a realistic estimation of the LOD based on a model exhibiting good generalization and an RMSE that is not underestimated. The training and test data were sampled randomly from each concentration level, ensuring equal representation across all levels. The mean of the RMSEs and the mean of the coefficients of determination (Q^2^) of all test runs were used to determine the number of PLS components sufficient for obtaining a low prediction error while avoiding overfitting. The minimum number of components resulting in a high mean Q^2^ and a low mean RMSE was selected. This procedure was performed 10 times in total, and the median number of latent variables was selected for LOD estimation. Subsequently, the entire dataset was employed for analysis. Equation (5) was used to derive the LOD from the predicted vs. the actual plot of both the PCR and the PLSR, while integrating leverage into the calculation. Both the RMSE and $s_{blank}$ were used for calculation. The LOQ was calculated likewise, but with a $k_{LOD}$ of 10.

## 4. Results and Discussion

### 4.1. Comparison of Different Approaches

When assessing approaches for estimating the LOD, determining the true value presents a challenge as there is no definitive or ‘superior’ answer. A lower LOD does not necessarily equate to a superior or worse method. Several authors have pointed out disparities (i.e., regression techniques, deviations, blank determination, k-factors, concentration ranges) in methods used to determine the detection limit for one-dimensional cases. This resulted in divergent LOD values [25,26,66,71,87,90] with discrepancies of up to a factor of six [91] or two orders of magnitude [67]. Our objective is to evaluate the performed approaches based on their dependencies and the underlying rationales. The calculated LODs for each substance using the respective methods are listed in Table 3. Raw data and code can be found in the Supplementary Materials section.

For our dataset, we noticed that using the RMSE mostly tends to lead to higher LOD values compared to employing $s_{blank}$ within the same approach (Table 3). Similar observations were made by other authors [26,28,91]. Initially, the RMSE is utilized to estimate $s_{blank}$; therefore, significant differences should not arise. We attribute this discrepancy to two main factors: either the univariate data lack linearity, or analyte-containing samples exhibit higher variances compared to blank samples. 

**Figure 4:**
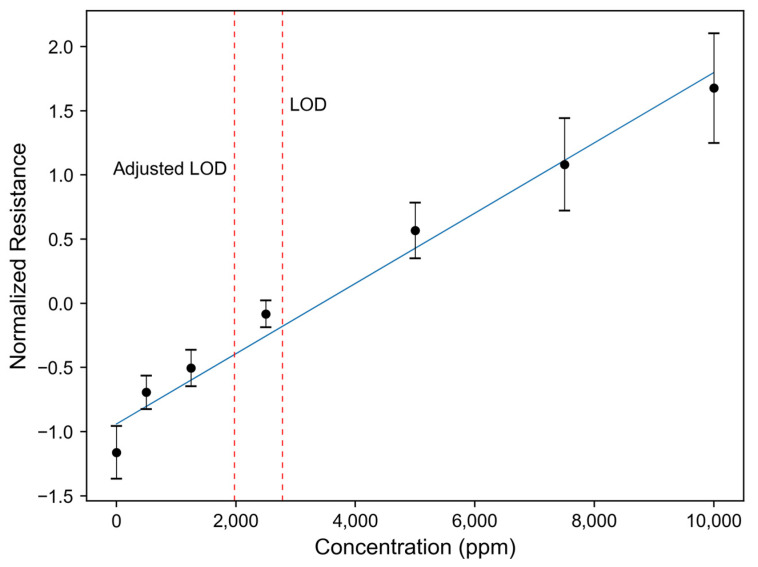
The normalized resistance over the concentration for sensor 24 for ethyl acetate. The calibration curve was calculated using OLS regression. Error bars correspond to the mean and standard deviation of the measurements per concentration level. The adjusted LOD is the LOD corrected by the difference between the intercept of the calibration curve and the mean sensor value of the blank.

The highest values were obtained via PCA II. It is evident that the unsupervised utilization of PC1 leads to higher values compared to the two multivariate regression techniques, as PC1 aims to account for the maximum variance within the data, disregarding its relationship to the target analyte. Consequently, this approach yields less linear and noisier univariate data, resulting in higher LODs. For PCA II and for single sensors, the mean sensor value of the blank measurements was lower than the y-intercept of the calibration curve, resulting in higher LOD values. Taking into account the difference between the intercept and the mean blank sensor value, an ‘adjusted’ LOD can be computed. The difference in the calculated LOD value is exemplarily illustrated for ethyl acetate in Figure 4 (for further substances see the Supplementary Figures and Tables) for the normalized resistance of sensor 24 at different concentrations. The adjusted LOD is about 800 ppm smaller than the LOD calculated directly using Equation (3). In the performed measurements, we observed a steeper increase in the signal in the lower concentration range for all substances. Referring to Equation (3), this means that, if the y-intercept is higher than the mean of the blank measurements, the formula yields to a higher LOD. This leads to the fact that a continuous presence of the linear region is assumed and, therefore, that the intercept approximately corresponds to the mean of the blank measurements. This should be kept in mind when using this equation. 

**Figure 5:**
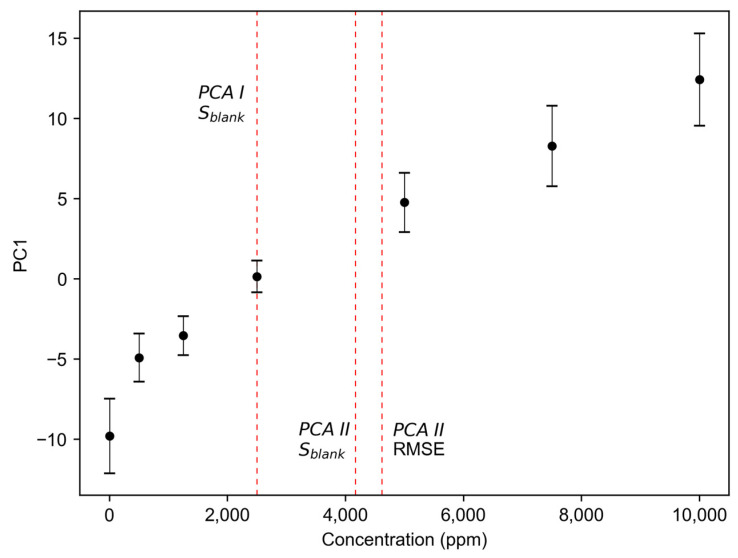
The calculated PC1 of sensor 24 over the concentration of ethyl acetate. Error bars correspond to the mean and standard deviation of the measurements per concentration level. The three different LOD values are shown, which were calculated using the respective method and standard deviation estimation.

In contrast, PCA I does not use the calibration curve and instead determines the lowest measured concentration surpassing the $y_{LOD}$ value. Because it therefore is not affected by the higher curve intercept and the mean of the actual values at low concentrations is mostly higher than the predicted values from the calibration curve, PCA I values tend to be lower. In conclusion, PCA II leads to higher results due to noisier and less linear univariate data, and PCA I is a good alternative if enough values close to the LOD are measured. Both methods are unsupervised and comparatively simple to carry out, but rely on a high explained variance of PC1, which was the case for our data (92–98%). The differences in the LOD values calculated using both methods are shown in Figure 5.

**Figure 6:**
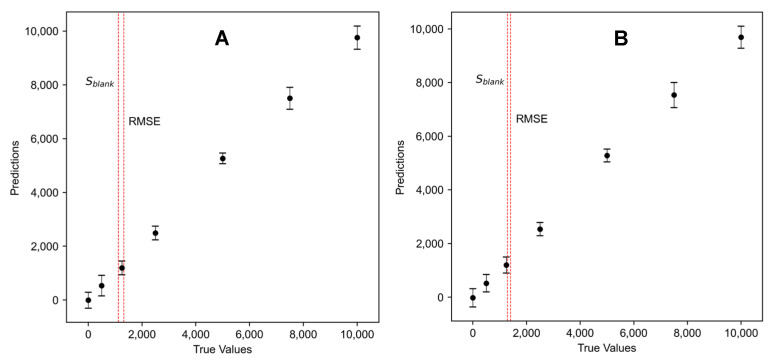
The predicted over tactual concentration for (**A**) the PLSR and (**B**) the PCR for ethyl acetate. Error bars correspond to the mean and standard deviation of the measurements per concentration level. Two different LOD values are shown, which were calculated using the annotated standard deviation estimation.

When comparing PCR and PLSR, the latter yields slightly lower LOD values. This discrepancy may arise because PCR’s latent variables primarily aim to explain the most variance, whereas PLSR focuses on minimizing residual differences in the regression model. As highlighted by [82], approaches employing multivariate regression techniques overlook the uncertainty inherent in estimated model parameters arising from the utilization of noisy calibration data. This can be observed by fluctuations in LODs when employing varying numbers of latent variables (in the case of using PLSR) and principal components (in the case of using PCR) or different numbers of folds in the cross validation to determine said variable numbers. Sufficient data is necessary to reduce this extra uncertainty and avoid generating excessively low LODs through this approach. Figure 6 shows the predicted concentration over the actual concentration for the PCR and PLSR model for ethyl acetate. Even if the LOD values calculated using the PLSR are slightly lower, all the calculated values are in a similar range and do not deviate as much as in the case using only PC1 (Figure 6).

In addition to the approaches detailed in Section 2 and summarized above, we explored two alternative methods that proved to be unsuccessful in our case for different reasons. The first method was devised for Near-Infrared Spectroscopy [28], which generates spectra and, thus, first-order data as well. Instead of utilizing a surrogate variable, they employed PLSR to determine the weight factors for each sensor, which could then be incorporated into the conventional one-dimensional LOD calculation. By combining these factors with the traditional IUPAC definition, they derived the following definition (Equation (7)) for ${\hat{c}}_{LOD}$ [28]:

(7) $${\hat{c}}_{LOD}=3*s_{blank(1\times J)}*b_{\left(J\times 1\right)}$$

Here $s_{blank}$ denotes the standard deviation vector of a blank, and $b_{\left(J\times 1\right)}$ represents the regression vector consisting of the PLSR coefficients. The method yielded relatively low LODs overall and resulted partly in negative LODs. We hypothesize that negative values may arise when sensors with negative PLSR weights are combined with a high standard deviation and positive weights are combined with a low standard deviation, causing the total sum to become negative. Consequently, we deem this method unsuitable for our application.

The second approach can be considered an intuitive approach as it is distinct from Currie’s proposed hypothesis testing. It was devised by Oleneva et al. [25] to estimate the LOD for multidimensional responses of sensor arrays. The fundamental concept is that, for samples falling below the LOD, the mean relative error (MRE) should exhibit a noticeable increase. Plotting the change in the averaged MRE values of the PLSR against analyte concentrations allows for LOD estimation. Oleneva et al. define the LOD as the concentration starting from which the change in the averaged MRE values are equal to or less than 1%.

In the course of applying this approach to our experiments, we encountered practical challenges that made the approach unsuitable for our data. While we did observe a similar curve pattern in the MRE to that reported in [25], the LOD definition did not align with the characteristics of our experiment. The authors, having measured 40 different concentrations, reached the 1% fluctuation threshold at concentrations above the LOD. In our scenario, where less, farther apart concentrations were measured more often, the MRE changes were significantly higher (e.g., 63% between the highest measured concentrations). Additionally, this approach demands the measurement of multiple concentrations below the LOD, and extrapolations, such as those utilized in other methods based on the calibration curve and hypothesis testing, are not possible. Furthermore, as emphasized by [92], at high concentrations of the analyte, other effects can introduce errors and corresponding fluctuations in the MRE, which typically should not impact the LOD. The approach has been shown to be more of a quantification tool and not suitable for a small set of concentrations with multiple replicas, such as that in our experimental setup.

### 4.2. Interday and Intraday Precision

As shown in Section 2, the LOD is directly dependent on the standard deviation of the blank or the residuals of the calibration curve. The larger these standard deviations and residuals, the higher the LOD. This holds true for any method, irrespective of the equation (see Table 2) applied. Consequently, the calculation of the standard deviation and residuals significantly impacts LOD determination. 

Considering our case used involves monitoring a multi-day beer-brewing process and examines a process beyond the laboratory environment, it was meaningful to use measurements of several days to reflect the limit that can be reached in practice. The signals of MOS sensors are, however, not as reproducible as one would anticipate for analytical instruments due to their drift behavior and the influence of the measurement history [6]. This is why the differences between the standard deviations of a single day for one sensor and all days for all sensors, as exemplarily shown for ethyl acetate in Figures S7 and S8 in the Supplementary Materials, were expected. It was observed that this effect was more prominent for some substances (e.g., 2-Phenylethanol) and less prominent for others (e.g., Diacetyl). In general, it can be said that the precision (described by the standard deviation) and thus the LOD can be improved if only the measurements of one day and one sensor are included. In turn, the variance can increase if measurements from all sensors and all days are included. The risk of a low variance caused by the inclusion of only one day is, however, the calculation of an LOD, which is ‘too low’ for a multi-day case.

The higher standard deviations within one concentration displayed in the PCA maps (Figure 5) are also due to the fact that no sensor selection was performed, but the data of all 62 gas sensors were considered for calculation. This again reflects our case best, although a sensor selection could have increased precision, as noisier and less informative sensors would have been completely removed. This can be especially concluded because individual sensors proved, with relative standard deviations of less than 10%, to be trustworthy over a wide concentration range. Nevertheless, even if the error bars are large, the information is included in the calculation of the LOD and a realistic estimate can therefore be expected. 

### 4.3. Comparisons of LODs and LOQs with Beer Composition and Feasibility for Beer-Brewing Application

For DMS, no LOD could be determined using the proposed methods as not enough quantitative data could be measured due to the sensor-poisoning effects of the substance. We observed signs of poisoning in the form of prolonged measurement times (until a steady-state signal was achieved) and a significantly extended baseline recovery period. Moreover, a sensitizing effect was noted, observed as an enhanced response to DMS on subsequent measurement days. Specifically, on the third day, a sample with only 0.05 ppm of DMS required a measurement phase of 20 min for the signal to stabilize, with an additional 2 h of purging with clean air needed to restore the baseline (as opposed to the usual 1–5 min, respectively). The observed poisoning effects of DMS are known disadvantages of MOS for sulfur-containing compounds such as SO_2_ and H_2_S [88,93,94,95,96]. Extrapolating the observations for DMS described before to the brewing process, this suggests that the eNose would likely respond to DMS at concentrations present in the process, but severe and irreversible poisoning effects are anticipated only at higher concentrations. DMS is the only VOC considered here that is technologically influenced before fermentation. Measuring times in the range of several minutes are probably too long to be able to respond with today’s technical means. Nevertheless, the analytical effort using the standard method of gas chromatography is very costly and cannot be achieved in many companies, which is why an alternative method for quality monitoring would be important in practice.

Sufficiently low LODs were attained solely for diacetyl, compared to the concentrations found in the maturation process (Table 1, Table 3). The end concentrations and OTVs are higher than the LODs calculated using a PLSR and the concentrations in the previous maturation process are even higher (Figure 1). Consequently, the LOQs for diacetyl were calculated. These values, in ppm, listed in the order of Table 3, are as follows: <0.5, 0.35, 0.43, 0.18, 0.16, 0.05 and 0.05, respectively. Subsequently, the estimated values for the LOD and the LOQ suggest potential for monitoring diacetyl online and in real-time in the beer-brewing process. These results are encouraging as diacetyl not only serves as an important off-flavor compound in beer but also functions as an indicator of the progress of maturation for other fermentation by-products in process control. Therefore, quality monitoring could be enhanced and process control improved.

It should be noted that changing the matrix from a 5% ethanolic solution to that of ‘real beer’ might affect the LODs, and the sufficient distinguishability of diacetyl (e.g., the specific fingerprint) needs to be checked. Additionally, MOS sensors are known for their intrinsic instability, so the LODs should not be interpreted as a hard threshold [22]. 

However, the other measured substances could not be monitored with an (this) electronic nose. Estimating the LODs made that apparent. For substances whose concentrations have decreased during the process, monitoring could still be possible in earlier stages of fermentation. This is also beneficial, as it could reduce the need for manual sampling.

## 5. Conclusions

Several methods have proven to be effective to estimate the LOD for substances measured with multidimensional sensor arrays in this study, and are preferable under different data scenarios and simplicity requirements. PCA, PCR, and PLSR were selected due to their widespread use in eNose applications, making them relevant for LOD estimation. The multivariate regression techniques yielded lower LOD values than using PCA. As the methods showed significant differences of up to a factor of eight, we strongly advise authors of eNose papers to provide detailed descriptions of their LOD and LOQ determination methods to enable readers to assess the methodology used and compare results cautiously. We suggest integrating leverage into LOD estimations to accommodate the differences in the selected concentrations. However, this does not replace the deliberate selection of concentrations near the LOD for calculation, particularly with MOS sensors, as they exhibit non-linearities in higher concentration ranges. We also recommend that the measured concentrations for a substance be recorded on consecutive days without interruption by measurements of other substances. This approach minimizes the influence of significant sensor drift on the scattering of the data points and on the results. 

The results suggest that eNose MOS technology holds promise for beer-brewing process control, although primarily for diacetyl monitoring. The diacetyl concentration is the most interesting of all VOCs because, in addition to its use in monitoring, it is already used today to control the fermentation and maturation process. This technology’s implementation in a new control technology is therefore conceivable. 

It is important to acknowledge that the LOD and LOQ values are specific to the particular eNose and sample composition utilized. Variations in factors like sensor array composition, sensor materials, and feature extraction methods will lead to different results. Additionally, the values obtained are influenced by the choice of calculation formula and multivariate data analysis techniques. 

In conclusion, this study has effectively bridged the gap between the concepts of LODs and eNoses, elucidating various methodologies and highlighting the strengths and weaknesses of each. The availability of standardized LOD estimation methods for eNoses is paramount for the advancement of the field. By making more LOD data publicly accessible, researchers can more readily identify suitable applications for eNose technology, accelerating its adoption across diverse domains.

Although LOD estimation with eNoses is not as precise and robust as that of typical analytical instruments, it can contribute to evaluating an eNose’s potential for a specific application. However, exceeding the LOD threshold alone does not ensure suitability, as the adequate differentiation of target substances and tresilience to the unintended environmental factors are also essential considerations.

## Figures and Tables

**Table 1:** The flavor and concentrations of key volatile compounds in beer (OTV = human odor threshold value).

Compound	Flavor in Beer	OTV in Bottom-Fermented Beer in ppm	Concentration in Beer in ppm
Diacetyl	‘butterscotch’, buttery	0.10–0.17 ^1,3,4^	0.01–0.12 ^3,9^
Isobutanol	alcoholic	10–100 ^5^	4–24 ^4,6^
2-Phenylethanol	rosy, sweetish	5–125 ^4,6^	4–51 ^6^
Ethyl acetate	solvent-like, fruity, sweet	25–30 ^2,4,5^	8–32 ^4,6^
Acetaldehyde	‘grassy’, green leaves	10–15 ^6^	2–20 ^4,6^
Dimethyl sulfide	herbaceous, celery-like	0.09–0.60 ^4,7,8^	0.01–0.14 ^7^

^1^—[43], ^2^—[41], ^3^—[52], ^4^—[53], ^5^—[48], ^6^—[33], ^7^—[54], ^8^—[55], ^9^—[39].

**Table 2:** Common equations for LOD estimation.

LOD Equation	Description	Employed Standard Deviation	References
$y_{LOD}={\bar{x}}_{b}+k_{LOD}\;*\;s_{b}$	- Calculates the sensor value that must be exceeded - Can only determine concentrations as LODs that have been measured	$s_{b}$	[27,63]
$c_{LOD}=\frac{k_{LOD}\;*\;s_{b}}{m}$	- Leverages the linear relationship between the concentration and the corresponding signal to determine concentrations as LODs that have not necessarily been directly measured	$s_{b}$	[58,67,68]
$c_{LOD}=\frac{k_{LOD}\;*\;s_{residual}}{m}$	- The curve deviation estimates $s_{b}$, for example, the $s_{residual}$. - Calibration samples need to be sufficiently representative of the test samples → residuals are comparable to instrumental noise - [23] found that for individual eNose sensors, the residual differences remain consistent throughout the measured range, thus suggesting that the $s_{residual}$ serves as a reliable estimate for noise	$s_{residual}$ (or intercept, slope)	[64,66,67,69]
$c_{LOD}=\frac{k_{LOD}\;*\;s_{residual}\;*\;\sqrt{\left(1+\frac{1}{n}+\frac{{\bar{c}}^{2}}{\sum {{(c}_{i}-\bar{c}}^{2})}\right)}}{m}$	- Incorporates leverage to account for additional uncertainties associated with the calibration curve based on the chosen concentration levels	$s_{residual}$	[72]

The abbreviations are as follows: $y_{LOD}$: the signal value of the corresponding LOD concentration; ${\bar{x}}_{b}$: the measured mean value of the analyte-free measurement as an estimate of its true mean $\mu_{b}$; $k_{LOD}$: the predefined factor defining error probabilities; $s_{b}$: the measured standard deviation of an analyte-free (blank) measurement as an estimate of the true standard deviation $\sigma_{b}$; m: the slope of the calibration curve (the analytical sensitivity); $s_{residual}$: the residual standard deviation, i.e., the RMSE (root mean square error); $s_{b}$ and $s_{residual}$ are defined as in Equations (2) and (4) in the main text.

**Table 3:** The limits of detection (LODs) calculated with different methods and utilized metrics. Values are in [ppm] in the sample solution, mostly rounded to two significant digits.

		PCA I ^1^	PCA II ^2^	PCA II ^2^	PCR	PCR	PLSR	PLSR
Standard Deviation		$s_{blank}$	$s_{blank}$	RMSE	$s_{blank}$	RMSE	$s_{blank}$	RMSE
Diacetyl	LOD	<0.10	0.14	0.17	0.07	0.07	0.02	0.02
Isobutanol	LOD	<500	1200	1600	300	460	210	320
2-Phenylethanol	LOD	<200	210	170	60	70	60	50
Ethyl acetate	LOD	<2500	4200	4600	1300	1400	1200	1300
Acetaldehyde	LOD	<6000	5200	9900	3500	3000	2800	2400

^1^ The smallest measured concentration whose mean of the corresponding principal component values (i.e. those of PC1) exceeds the calculated principal component value from Equation (1); ^2^ using the calibration curve and Equation (5) (see calculating LOD and LOQ).

## Data Availability

The original contributions presented in the study are included in the Supplementary Materials; further inquiries can be directed to the corresponding author/s.

## Supplementary Materials

The following supporting information can be downloaded at: https://www.mdpi.com/article/10.3390/s24113520/s1, Figure S1: The mass flow controllers (1) allow to mix dry air and humid air (2) for controlling the humidity of the carrier gas directed to the electronic nose (4). The sample vessel (3) is connected to the electronic nose by three tubes. These are used to remove ambient air in the vessel and to direct sample air to the sensor. The notebook (5) was used to control the system and read out the data; Figure S2: Compressed air from a gas cylinder was used as clean air as carrier gas and for sensor recovery. Mass flow controllers were used to control humidity by mixing dry and humid air. The electronic nose is connected by three tubes with the sample: one tube allows the clean air to be fed into the sample vessel; A second tube is used to direct the sample air to the sensors; the third tube directs the air out of the device without washing over the sensors—this allows ambient air to be removed from the sample vessel; Figure S3: The first principal component (PC1) plotted over the concentration levels of diacetyl. PC1 explained 99.59% of the variance of the data. The error bars correspond to the mean and standard deviation of the measurements per concentration level; Figure S4: The first principal component (PC1) plotted over the concentration levels of diacetyl. PC1 explained 99.59% of the variance of the data. The error bars correspond to the mean and standard deviation of the measurements per concentration level. The calibration curve was calculated using OLS; Figure S5: Predicted and true values of the concentration of diacetyl for the PCR model with 10 components and a Q2 of 0.9973 and an RMSE of 0.018; Figure S6: Predicted and true values of the concentration of diacetyl for the PLSR model with 11 components and a Q2 of 0.9999 and an RMSE of 0.004; Figure S7: Exemplary feature values for sensor 1 over the concentration levels of ethyl acetate for a single measurement day. The error bars correspond to the mean and standard deviation of the measurements per concentration level; Figure S8: The first principal component (PC1) plotted over the concentration levels of ethyl acetate. PC1 explained 98.95% of the variance of the data. The error bars correspond to the mean and standard deviation of the measurements per concentration level; Figure S9: The first principal component (PC1) plotted over the concentration levels of ethyl acetate. PC1 explained 98.95% of the variance of the data. The error bars correspond to the mean and standard deviation of the measurements per concentration level. The calibration curve was calculated using OLS; Figure S10: Predicted and true values of the concentration of ethyl acetate for the PCR model with 19 components and a Q2 of 0.9886 and an RMSE of 371.897; Figure S11: Predicted and true values of the concentration of ethyl acetate for the PLSR model with 7 components and a Q2 of 0.9899 and an RMSE of 349.168; Figure S12: The first principal component (PC1) plotted over the concentration levels of 2-Phenylethanol. PC1 explained 97.91% of the variance of the data. The error bars correspond to the mean and standard deviation of the measurements per concentration level; Figure S13: The first principal component (PC1) plotted over the concentration levels of 2-Phenylethanol. PC1 explained 98.95% of the variance of the data. The error bars correspond to the mean and standard deviation of the measurements per concentration level. The calibration curve was calculated using OLS; Figure S14: Predicted and true values of the concentration of 2-Phenylethanol for the PCR model with 19 components and a Q2 of 0.9406 and an RMSE of 17.729; Figure S15: Predicted and true values of the concentration of 2-Phenylethanol for the PLSR model with 8 components and a Q2 of 0.9736 and an RMSE of 11.819; Figure S16: The first principal component (PC1) plotted over the concentration levels of isobutanol. PC1 explained 98.44% of the variance of the data. The error bars correspond to the mean and standard deviation of the measurements per concentration level; Figure S17: The first principal component (PC1) plotted over the concentration levels of isobutanol. PC1 explained 98.44% of the variance of the data. The error bars correspond to the mean and standard deviation of the measurements per concentration level. The calibration curve was calculated using OLS; Figure S18: Predicted and true values of the concentration of isobutanol for the PCR model with 15 components and a Q2 of 0.9717 and an RMSE of 119.021; Figure S19: Predicted and true values of the concentration of isobutanol for the PLSR model with 7 components and a Q2 of 0.9815 and an RMSE of 96.189; Figure S20: The first principal component (PC1) plotted over the concentration levels of acetaldehyde. PC1 explained 93.47% of the variance of the data. The error bars correspond to the mean and standard deviation of the measurements per concentration level. A high standard deviation of the measurements can be observed; Figure S21: The first principal component (PC1) plotted over the concentration levels of acetaldehyde. PC1 explained 93.47% of the variance of the data. The error bars correspond to the mean and standard deviation of the measurements per concentration level. The calibration curve was calculated using OLS; Figure S22: Predicted and true values of the concentration of acetaldehyd for the PCR model with 16 components and a Q2 of 0.9694 and an RMSE of 848.776; Figure S23: Predicted and true values of the concentration of acetaldehyd for the PCR model with 9 components and a Q2 of 0.9799 and an RMSE of 688.082; Table S1: Number of measurements conducted for each concentration level of diacetyl. The concentration of 0.00 ppm comprises the measurements of the 5% *v*/*v* ethanol-water solution; Table S2: Number of measurements conducted for each concentration level of ethyl acetate. The concentration of 0 ppm comprises the measurements of the 5% *v*/*v* ethanol-water solution; Table S3: Number of measurements conducted for each concentration level of 2-Phenylethanol. The concentration of 0 ppm comprises the measurements of the 5% *v*/*v* ethanol-water solution; Table S4: Number of measurements conducted for each concentration level of isobutanol. The concentration of 0 ppm comprises the measurements of the 5% *v*/*v* ethanol-water solution; Table S5: Number of measurements conducted for each concentration level of acetaldehyde. The concentration of 0 ppm comprises the measurements of the 5% *v*/*v* ethanol-water solution.

## References

[1] Boqué R., Faber N.M., Rius F. (2000). Detection limits in classical multivariate calibration models. Anal. Chim. Acta.

[2] Allegrini F., Olivieri A.C. (2014). IUPAC-consistent approach to the limit of detection in partial least-squares calibration. Anal. Chem..

[3] Alsaedi B.S., McGraw C.M., Schaerf T.M., Dillingham P.W. (2020). Multivariate limit of detection for non-linear sensor arrays. Chemom. Intell. Lab. Syst..

[4] Persaud K., Dodd G. (1982). Analysis of discrimination mechanisms in the mammalian olfactory system using a model nose. Nature.

[5] John A.T., Murugappan K., Nisbet D.R., Tricoli A. (2021). An Outlook of Recent Advances in Chemiresistive Sensor-Based Electronic Nose Systems for Food Quality and Environmental Monitoring. Sensors.

[6] Karakaya D., Ulucan O., Turkan M. (2020). Electronic Nose and Its Applications: A Survey. Int. J. Autom. Comput..

[7] Seesaard T., Goel N., Kumar M., Wongchoosuk C. (2022). Advances in gas sensors and electronic nose technologies for agricultural cycle applications. Comput. Electron. Agric..

[8] Sekhar P.K., Brosha E.L., Mukundan R., Garzon F. (2010). Chemical Sensors for Environmental Monitoring and Homeland Security. Electrochem. Soc. Interface.

[9] Loutfi A., Coradeschi S., Mani G.K., Shankar P., Rayappan J.B.B. (2015). Electronic noses for food quality: A review. J. Food Eng..

[10] Khorramifar A., Karami H., Lvova L., Kolouri A., Łazuka E., Piłat-Rożek M., Łagód G., Ramos J., Lozano J., Kaveh M. (2023). Environmental Engineering Applications of Electronic Nose Systems Based on MOX Gas Sensors. Sensors.

[11] Bhowmik B., Gupta R.K. (2023). Chemical sensors for e-nose: An effective route for disease diagnosis. Nanotechnology-Based E-Noses: Fundamentals and Emerging Applications.

[12] Wilson A.D., Forse L.B. (2023). Potential for Early Noninvasive COVID-19 Detection Using Electronic-Nose Technologies and Disease-Specific VOC Metabolic Biomarkers. Sensors.

[13] Wörner J., Moelleken M., Dissemond J., Pein-Hackelbusch M. (2023). Supporting wound infection diagnosis: Advancements and challenges with electronic noses. Front. Sens..

[14] Park S.Y., Kim Y., Kim T., Eom T.H., Kim S.Y., Jang H.W. (2019). Chemoresistive materials for electronic nose: Progress, perspectives, and challenges. InfoMat.

[15] Chen Z., Chen Z., Song Z., Ye W., Fan Z. (2019). Smart gas sensor arrays powered by artificial intelligence. J. Semicond..

[16] Mitrovics J. (2004). Auswerteverfahren für Gassensorarrays. Ph.D. Thesis.

[17] Wilson A.D. (2012). Review of Electronic-nose Technologies and Algorithms to Detect Hazardous Chemicals in the Environment. Procedia Technol..

[18] Jurs P.C., Bakken G.A., McClelland H.E. (2000). Computational methods for the analysis of chemical sensor array data from volatile analytes. Chem. Rev..

[19] Frank Röck U.W., Sommer K.-D., Puente León F. (2007). Elektronische Nase und Signalgewinnung. Informationsfusion in der Mess- und Sensortechnik.

[20] Peveler W.J., Yazdani M., Rotello V.M. (2016). Selectivity and Specificity: Pros and Cons in Sensing. ACS Sens..

[21] Subandri M.A., Sarno R. (2019). E-Nose sensor array optimization based on volatile compound concentration data. J. Phys. Conf. Ser..

[22] Burgués J., Jiménez-Soto J.M., Marco S. (2018). Estimation of the limit of detection in semiconductor gas sensors through linearized calibration models. Anal. Chim. Acta.

[23] Kang N.K., Jun T.S., La D.-D., Oh J.H., Cho Y.W., Kim Y.S. (2010). Evaluation of the limit-of-detection capability of carbon black-polymer composite sensors for volatile breath biomarkers. Sens. Actuators B Chem..

[24] Parastar H., Kirsanov D. (2020). Analytical Figures of Merit for Multisensor Arrays. ACS Sens..

[25] Oleneva E., Khaydukova M., Ashina J., Yaroshenko I., Jahatspanian I., Legin A., Kirsanov D. (2019). A Simple Procedure to Assess Limit of Detection for Multisensor Systems. Sensors.

[26] Rahman H., Rahman M.M. (2015). Estimation of Limit of Detection (LOD), Limit of Quantification (LOQ) and Machine Standardization by Gas Chromatography. Ann. Bangladesh Agric..

[27] Sanchez J. (2018). Estimating Detection Limits in Chromatography from Calibration Data: Ordinary Least Squares Regression vs. Weighted Least Squares. Separations.

[28] Ostra M., Ubide C., Vidal M., Zuriarrain J. (2008). Detection limit estimator for multivariate calibration by an extension of the IUPAC recommendations for univariate methods. Analyst.

[29] Gonzalez Viejo C., Fuentes S., Godbole A., Widdicombe B., Unnithan R.R. (2020). Development of a low-cost e-nose to assess aroma profiles: An artificial intelligence application to assess beer quality. Sens. Actuators B Chem..

[30] Santos J.P., Lozano J. Real time detection of beer defects with a hand held electronic nose. Proceedings of the 2015 10th Spanish Conference on Electron Devices (CDE), Aranjuez.

[31] Ghasemi-Varnamkhasti M., Mohtasebi S.S., Siadat M., Lozano J., Ahmadi H., Razavi S.H., Dicko A. (2011). Aging fingerprint characterization of beer using electronic nose. Sens. Actuators B Chem..

[32] Andrés-Iglesias C., Montero O., Sancho D., Blanco C.A. (2015). New trends in beer flavour compound analysis. J. Sci. Food Agric..

[33] European Brewery Convention (2000). Fermentation and Maturation.

[34] Trelea I.C., Landaud S., Latrille E., Corrieu G. (2002). Prediction of Confidence Limits for Diacetyl Concentration during Beer Fermentation. J. Am. Soc. Brew. Chem..

[35] Kurz T., Fellner M., Becker T., Delgado A. (2001). Observation and Control of the Beer Fermentation Using Cognitive Methods. J. Inst. Brew..

[36] Tritz F., Fosso P., Brandstetter T., Rühe J. Development of a mobile detection system for the quantitative determination of vicinal diketones from green beer [Poster]. Proceedings of the EBC Congress.

[37] Querol A., Fleet G. (2006). Yeasts in Food and Beverages: With 30 Tables.

[38] Krogerus K., Gibson B.R. (2013). 125 th Anniversary Review: Diacetyl and its control during brewery fermentation. J. Inst. Brew..

[39] Brányik T., Vicente A.A., Dostálek P., Teixeira J.A. (2008). A Review of Flavour Formation in Continuous Beer Fermentations*. J. Inst. Brew..

[40] Sakamoto K., Konings W.N. (2003). Beer spoilage bacteria and hop resistance. Int. J. Food Microbiol..

[41] Kobayashi M., Nagahisa K., Shimizu H., Shioya S. (2006). Simultaneous control of apparent extract and volatile compounds concentrations in low-malt beer fermentation. Appl. Microbiol. Biotechnol..

[42] Geiger E., Piendl A. (1975). Technological Influences on the Formation of 2-Phenylethanol during Fermentation. Proc. Annu. Meet.-Am. Soc. Brew. Chem..

[43] Olaniran A.O., Hiralal L., Mokoena M.P., Pillay B. (2017). Flavour-active volatile compounds in beer: Production, regulation and control. J. Inst. Brew..

[44] Gee D.A., Ramirez W.F. (1994). A flavour model for beer fermentation. J. Inst. Brew..

[45] Kobayashi M., Shimizu H., Shioya S. (2008). Beer volatile compounds and their application to low-malt beer fermentation. J. Biosci. Bioeng..

[46] Scheuren H., Baldus M., Methner F.-J., Dillenburger M. (2016). Evaporation behaviour of DMS in an aqueous solution at infinite dilution—A review. J. Inst. Brew..

[47] Deutsches Institut für Normung (2021). Sudhausanlagen in Brauereien, Brewhouse Plants—Minimum Specifications.

[48] Annemüller G., Manger H.-J., Lietz P. (2013). Die Hefe in der Brauerei: Hefemanagement, Kulturhefe—Hefereinzucht, Hefepropagation im Bierherstellungsprozess.

[49] Pajunen E., Enari T.-M. (1978). Accelerated lagering and maturation. Monograph V. E.B.C. Fermentation and Storage Symposium.

[50] Hegarty P.K., Parsons R., Bamforth C.W., Molzahn S.W. (1995). Phenyl ethanol—A factor determining lager character. European Brewery Convention: Proceedings of the 25th Congress.

[51] Busch-Stockfisch M., Sensorik Kompakt (2002). In der Produktentwicklung und Qualitätssicherung.

[52] Saison D., de Schutter D.P., Uyttenhove B., Delvaux F., Delvaux F.R. (2009). Contribution of staling compounds to the aged flavour of lager beer by studying their flavour thresholds. Food Chem..

[53] Harrison G.A.F. (1970). The flavour of beer—A review. J. Inst. Brew..

[54] Grigsby J.H., Palamand S.R. (1977). A Colorimetric Procedure for the Measurement of Dimethyl Sulfide in Water, Wort, and Beer. J. Am. Soc. Brew. Chem..

[55] Brown D., Clapperton J., MeilGaard M., Moll M. (1978). Flavor Thresholds of Added Substances. J. Am. Soc. Brew. Chem..

[56] Dillingham P.W., Alsaedi B.S.O., Granados-Focil S., Radu A., McGraw C.M. (2020). Establishing Meaningful Limits of Detection for Ion-Selective Electrodes and Other Nonlinear Sensors. ACS Sens..

[57] Currie L.A. (1995). Nomenclature in evaluation of analytical methods including detection and quantification capabilities: IUPAC Recommendations 1995. Pure Appl. Chem..

[58] MacDougall D., Amore F.J., Cox G.V., Crosby D.G., Estes F.L., Freeman D.H., Gibbs W.E., Gordon G.E., Keith L.H., Crummett W.B. (1980). Guidelines for data acquisition and data quality evaluation in environmental chemistry. Anal. Chem..

[59] (1997). ISO 11843-1.

[60] International Council for Harmonisation of Technical Requirements for Pharmaceuticals for Human Use (2022). Validation of Analytical Procedures: Q2 (R2).

[61] Analytical Methods Committee (1987). Recommendations for the definition, estimation and use of the detection limit. Analyst.

[62] Henry W. (1803). Experiments on the quantity of gases absorbed by water, at different temperatures, and under different pressures. Philos. Trans. R. Soc..

[63] Currie L.A. (1968). Limits for qualitative detection and quantitative determination. Application to radiochemistry. Anal. Chem..

[64] Miller J., Miller J. (2010). Statistics and Chemometrics for Analytical Chemistry.

[65] Feng Y., Tian X., Chen Y., Wang Z., Xia J., Qian J., Zhuang Y., Chu J. (2021). Real-time and on-line monitoring of ethanol fermentation process by viable cell sensor and electronic nose. Bioresour. Bioprocess..

[66] Bernal E., Guo X. (2014). Limit of Detection and Limit of Quantification Determination in Gas Chromatography. Advances in Gas Chromatography.

[67] Mocak J., Bond A.M., Mitchell S., Scollary G. (1997). A statistical overview of standard (IUPAC and ACS) and new procedures for determining the limits of detection and quantification: Application to voltammetric and stripping techniques (Technical Report). Pure Appl. Chem..

[68] Long G.L., Winefordner J.D. (1983). Limit of detection. A closer look at the IUPAC definition. Anal. Chem..

[69] Olivieri A.C., Faber N.M., Ferré J., Boqué R., Kalivas J.H., Mark H. (2006). Uncertainty estimation and figures of merit for multivariate calibration (IUPAC Technical Report). Pure Appl. Chem..

[70] Del Río Bocio F.J., Riu J., Boqué R., Rius F.X. (2003). Limits of detection in linear regression with errors in the concentration. J. Chemom..

[71] Zorn M.E., Gibbons R.D., Sonzogni W.C. (1999). Evaluation of Approximate Methods for Calculating the Limit of Detection and Limit of Quantification. Environ. Sci. Technol..

[72] Olivieri A.C. (2015). Practical guidelines for reporting results in single- and multi-component analytical calibration: A tutorial. Anal. Chim. Acta.

[73] Taoping L., Lihua G., Mou W., Chen S., Di W., Hao D., Jingdong C., Weiwei W. (2023). Review on Algorithm Design in Electronic Noses: Challenges, Status and Trends. Intell. Comput..

[74] Güntner A.T., Koren V., Chikkadi K., Righettoni M., Pratsinis S.E. (2016). E-Nose Sensing of Low-ppb Formaldehyde in Gas Mixtures at High Relative Humidity for Breath Screening of Lung Cancer?. ACS Sens..

[75] Qin P., Okur S., Jiang Y., Heinke L. (2022). A MOF-based electronic nose for carbon dioxide sensing with enhanced affinity and selectivity by ionic-liquid embedment. J. Mater. Chem. A.

[76] Berna A.Z., Trowell S., Cynkar W., Cozzolino D. (2008). Comparison of metal oxide-based electronic nose and mass spectrometry-based electronic nose for the prediction of red wine spoilage. J. Agric. Food Chem..

[77] Arroyo P., Meléndez F., Suárez J.I., Herrero J.L., Rodríguez S., Lozano J. (2020). Electronic Nose with Digital Gas Sensors Connected via Bluetooth to a Smartphone for Air Quality Measurements. Sensors.

[78] Yang W., Wan P., Jia M., Hu J., Guan Y., Feng L. (2015). A novel electronic nose based on porous In_2_O_3_ microtubes sensor array for the discrimination of VOCs. Biosens. Bioelectron..

[79] Gu S., Wang J., Wang Y. (2019). Early discrimination and growth tracking of *Aspergillus* spp. contamination in rice kernels using electronic nose. Food Chem..

[80] Vlasov Y., Legin A., Rudnitskaya A., Di Natale C., D’Amico A. (2005). Nonspecific sensor arrays (“electronic tongue”) for chemical analysis of liquids (IUPAC Technical Report). Pure Appl. Chem..

[81] Kurita T. (2020). Principal Component Analysis (PCA). Int. J. Comput. Vis..

[82] Burgués J., Marco S. (2018). Multivariate estimation of the limit of detection by orthogonal partial least squares in temperature-modulated MOX sensors. Anal. Chim. Acta.

[83] Singh A. (1993). Multivariate decision and detection limits. Anal. Chim. Acta.

[84] Wold S., Sjöström M., Eriksson L. (2001). PLS-regression: A basic tool of chemometrics. Chemom. Intell. Lab. Syst..

[85] Ortiz M., Sarabia L., Herrero A., Sánchez M., Sanz M., Rueda M., Giménez D., Meléndez M. (2003). Capability of detection of an analytical method evaluating false positive and false negative (ISO 11843) with partial least squares. Chemom. Intell. Lab. Syst..

[86] International Conference on Harmonisation of Technical Requirements for Registration of Pharmaceuticals for Human Use (2005). ICH Harmonised Tripartite Guideline: Validation of Analytical Procedures: Text and Methodology Q2(R1).

[87] Shrivastava A., Gupta V. (2011). Methods for the determination of limit of detection and limit of quantitation of the analytical methods. Chron. Young Sci..

[88] Arshak K., Moore E., Lyons G.M., Harris J., Clifford S. (2004). A review of gas sensors employed in electronic nose applications. Emerald.

[89] Wasilewski T., Migoń D., Gębicki J., Kamysz W. (2019). Critical review of electronic nose and tongue instruments prospects in pharmaceutical analysis. Anal. Chim. Acta.

[90] Montville D., Voigtman E. (2003). Statistical properties of limit of detection test statistics. Talanta.

[91] Sanagi M.M., Ling S.L., Nasir Z., Hermawan D., Wan Ibrahim W.A., Naim A.A. (2009). Comparison of Signal-to-Noise, Blank Determination, and Linear Regression Methods for the Estimation of Detection and Quantification Limits for Volatile Organic Compounds by Gas Chromatography. J. AOAC Int..

[92] Poggialini F., Legnaioli S., Campanella B., Cocciaro B., Lorenzetti G., Raneri S., Palleschi V. (2023). Calculating the Limits of Detection in Laser-Induced Breakdown Spectroscopy: Not as Easy as It Might Seem. Appl. Sci..

[93] Das S., Chakraborty S., Parkash O., Kumar D., Bandyopadhyay S., Samudrala S.K., Sen A., Maiti H.S. (2008). Vanadium doped tin dioxide as a novel sulfur dioxide sensor. Talanta.

[94] Berger F., Fromm M., Chambaudet A., Planade R. (1997). Tin dioxide-based gas sensors for SO_2_ detection: A chemical interpretation of the increase in sensitivity obtained after a primary detection. Sens. Actuators B Chem..

[95] Deshmukh S., Bandyopadhyay R., Bhattacharyya N., Pandey R.A., Jana A. (2015). Application of electronic nose for industrial odors and gaseous emissions measurement and monitoring—An overview. Talanta.

[96] Chai H., Zheng Z., Liu K., Xu J., Wu K., Luo Y., Liao H., Debliquy M., Zhang C. (2022). Stability of Metal Oxide Semiconductor Gas Sensors: A Review. IEEE Sens. J..

