# Supplementary material for: Methods for Estimating the Detection and Quantification Limits of Key Substances in Beer Maturation with Electronic Noses
Source: Sensors (Basel). 2024 May 30;24(11):3520. doi: 10.3390/s24113520 (PMC11175341; doi:10.3390/s24113520)
Supplement: Supplementary file 1 [file sensors-24-03520-s001.zip › Code.html]

Code


This example shows the data for acetaldehyde

In [10]:

```
#Read csv file with features of substance
import pandas as pd

directory = 'INSERT DIRECTORY'

#First column is for indexes
features = pd.read_csv(directory, sep="\t", index_col=0)
```

In [11]:

```
#Exclude temperature & humidity sensor and information about the day
features = features.drop('Temperature', axis=1)
features = features.drop('Humidity Difference', axis=1)
features = features.drop('Day', axis=1)
```

In [12]:

```
#Display the 62 features for all measurements
features
```

Out[12]:

|  | 0 | 1 | 2 | 3 | 4 | 5 | 6 | 7 | 8 | 9 | ... | 53 | 54 | 55 | 56 | 57 | 58 | 59 | 60 | 61 | Concentration |
| --- | --- | --- | --- | --- | --- | --- | --- | --- | --- | --- | --- | --- | --- | --- | --- | --- | --- | --- | --- | --- | --- |
| 0 | -15414.933333 | -18689.766667 | -6075.133333 | -6299.7 | -5185.333333 | -5543.366667 | 2357.166667 | 1999.166667 | -5240.633333 | -3232.966667 | ... | -15054.666667 | -17127.766667 | -14964.366667 | -15731.800000 | -14458.333333 | -12710.933333 | -14868.000000 | 2.031147e+04 | 1.199987e+04 | 1000.0 |
| 1 | -31579.133333 | -30115.766667 | -10357.233333 | -10122.9 | -10448.633333 | -10742.266667 | -2068.933333 | -2118.633333 | -8679.833333 | -5463.166667 | ... | -22324.466667 | -25981.166667 | -27234.866667 | -23750.800000 | -22456.433333 | -27059.333333 | -26662.500000 | -4.810843e+04 | -3.533303e+04 | 1000.0 |
| 2 | -35462.333333 | -35657.466667 | -11825.733333 | -11338.1 | -11443.133333 | -11851.566667 | -2666.933333 | -1340.233333 | -8159.233333 | -5319.566667 | ... | -23017.466667 | -29283.866667 | -26761.666667 | -21870.900000 | -28322.133333 | -31144.833333 | -23881.900000 | -2.981833e+03 | -6.346483e+04 | 1000.0 |
| 3 | -23613.333333 | -26278.966667 | -8468.233333 | -9287.7 | -3791.033333 | -3512.666667 | 10541.666667 | 10696.866667 | -1945.333333 | -368.766667 | ... | -10073.966667 | -9204.966667 | -8459.366667 | -9199.400000 | -10403.933333 | -6885.933333 | -5762.400000 | 1.098370e+05 | 9.511247e+04 | 2000.0 |
| 4 | -28928.533333 | -31346.566667 | -10811.933333 | -11041.2 | -7952.033333 | -9997.366667 | 4355.566667 | 4067.866667 | -6547.433333 | -2876.566667 | ... | -22255.766667 | -22931.966667 | -22609.966667 | -21363.300000 | -21685.033333 | -21836.433333 | -22170.600000 | 4.585537e+04 | 5.037567e+04 | 2000.0 |
| ... | ... | ... | ... | ... | ... | ... | ... | ... | ... | ... | ... | ... | ... | ... | ... | ... | ... | ... | ... | ... | ... |
| 100 | 145203.933333 | 148993.466667 | 27564.666667 | 31059.7 | 56307.566667 | 58206.166667 | 72561.866667 | 72951.933333 | 29806.800000 | 31767.100000 | ... | 99756.000000 | 94635.833333 | 93545.333333 | 88659.966667 | 87896.233333 | 108120.533333 | 107247.033333 | 1.290786e+06 | 1.353217e+06 | 9000.0 |
| 101 | 129621.533333 | 132768.766667 | 22773.966667 | 23577.4 | 49836.866667 | 48231.166667 | 67353.166667 | 66695.633333 | 27091.000000 | 25483.200000 | ... | 78886.400000 | 73234.433333 | 73072.333333 | 66713.366667 | 66039.433333 | 86519.433333 | 83760.533333 | 1.200376e+06 | 1.276475e+06 | 9000.0 |
| 102 | 6887.433333 | 6045.666667 | 4565.066667 | 4845.8 | 6077.366667 | 6784.766667 | 5651.966667 | 4163.033333 | 3838.800000 | 4432.000000 | ... | 16623.900000 | 18093.733333 | 17403.333333 | 18711.766667 | 18745.833333 | 20696.833333 | 22963.233333 | 1.249697e+04 | 9.307600e+03 | 0.0 |
| 103 | 766.333333 | -1595.833333 | -680.033333 | -476.9 | -235.333333 | -2282.833333 | -1103.633333 | 202.033333 | -120.900000 | -145.000000 | ... | -2813.300000 | -1834.966667 | -2259.266667 | -1822.733333 | -1568.566667 | -1535.866667 | -2754.666667 | 1.427217e+04 | -3.416400e+03 | 0.0 |
| 104 | -7653.766667 | -4449.833333 | -3885.033333 | -4368.9 | -5842.033333 | -4501.933333 | -4548.333333 | -4365.066667 | -3717.900000 | -4287.000000 | ... | -13810.600000 | -16258.766667 | -15144.066667 | -16889.033333 | -17177.266667 | -19160.966667 | -20208.566667 | -2.676913e+04 | -5.891200e+03 | 0.0 |

105 rows × 63 columns

In [13]:

```
'''
#only for Isobutanol, the highest concentration was not used as it was too high
values = [0,1000,2000,250,500]
features = features.loc[features.iloc[:,-1].isin(values)]
features = features.reset_index(drop=True)
'''
```

Out[13]:

```
'\n#only for Isobutanol\nvalues = [0,1000,2000,250,500]\nfeatures = features.loc[features.iloc[:,-1].isin(values)]\nfeatures = features.reset_index(drop=True)\n'
```

In [14]:

```
#Standardize features with z-scores
normalized_features = features.copy()
normalized_features.iloc[:,:-1] = (normalized_features.iloc[:,:-1]-normalized_features.iloc[:,:-1].mean())/normalized_features.iloc[:,:-1].std()
```

In [15]:

```
#Look at the concentration levels and number of samples per level
normalized_features.iloc[:,-1].value_counts()
```

Out[15]:

```
0.0        16
500.0      13
6000.0     13
1000.0     12
4000.0     12
2000.0     11
9000.0     10
12000.0     9
15000.0     9
Name: Concentration, dtype: int64
```

In [16]:

```
#Calculation of the effective leverage

#number of samples
n = len(features)

#effective leverage calculation
cmean = normalized_features.iloc[:,-1].mean()
sumres = 0
#calculate the sum of the residuals
for c in normalized_features.iloc[:,-1]:
    res = (c-cmean)**2
    sumres = sumres+res

leverage = (cmean**2)/sumres
leverage = leverage+(1/n) #1/n added to the leverage to get the effective leverage
```

In [17]:

```
#get an idea of the PC1 vs. concentration plot
from sklearn.decomposition import PCA
import matplotlib.pyplot as plt

#fit PCA
pca_ = PCA(n_components=2)
principalComponents_ = pca_.fit_transform(normalized_features.iloc[:,:-1])
principal_Df = pd.DataFrame(data = principalComponents_, columns = ['principal component 1', 'principal component 2'])

#plot error bars
principal_Df["Concentration"] = normalized_features.iloc[:,-1]
plt.errorbar(principal_Df.groupby('Concentration').mean().index, principal_Df.groupby('Concentration').mean().iloc[:,0], yerr=principal_Df.groupby('Concentration').std().iloc[:,0], marker='o', capsize=3, markersize="4", color='black', ls='none', linewidth=0.5)


# display plot
plt.xlabel('Concentration [ppm]')
plt.ylabel('PC1')
plt.show()

#Print the explained variance of the first component
print("Explained variance of first PC: ", pca_.explained_variance_ratio_[0])
```

```
Explained variance of first PC:  0.9346590025991751
```

In [18]:

```
#define the k for the LOD estiamtion

#set kLOD to 3.3 (or to 2*t(1-α,v) for smaller number of samples)
kLOD = 3.72 
#kLOD = 4.03 #for Diacetyl due to smaller sample size
#kLOD = 10 #for LOQ
```

In [20]:

```
############################## Methods based on pseudo univariate calculation with PCA ##############################
import numpy as np
from scipy import stats
from sklearn.metrics import mean_squared_error
import math

#calculation of yLOD

#Only consider the components of the samples with a concentration of zero
values = [0]

#calculate the mean and standard deviation of the first principal component for the blanks
meanblank = principal_Df[normalized_features.iloc[:,-1].isin(values)]["principal component 1"].mean()
stdblank = principal_Df[normalized_features.iloc[:,-1].isin(values)]["principal component 1"].std()

yLOD = meanblank+(kLOD*stdblank)
print("yLOD for PC1 vs. concentration plot: ", yLOD)
#calculate the mean PC-value of every concentration level
new_df = pd.DataFrame()
new_df["PC1"] = principal_Df["principal component 1"]
new_df["Concentration"] = normalized_features.iloc[:,-1]
print(new_df.groupby(new_df.iloc[:,-1]).mean())

#Calculate the calibration curve and plot the function
y = principal_Df.loc[:, 'principal component 1']
x = normalized_features.iloc[:,-1]
slope, intercept, r, p, std_err = stats.linregress(x, y)

def myfunc(x):
  return slope * x + intercept

mymodel = list(map(myfunc, x))
plt.xlabel('Concentration [ppm]')
plt.ylabel('PC1')

#plot the error bars
principal_Df["Concentration"] = normalized_features.iloc[:,-1]
plt.errorbar(principal_Df.groupby('Concentration').mean().index, principal_Df.groupby('Concentration').mean().iloc[:,0], yerr=principal_Df.groupby('Concentration').std().iloc[:,0], marker='o', capsize=3, markersize="4", color='black', ls='none', linewidth=0.5)
plt.plot(x, mymodel, linewidth=0.5)
plt.show() 
plt.close()

#calculation of cLOD

#The slope of the calibration curve
m = slope

#First use the function of the sklearn library to calculate the RMSE
#remark: classical calibration is used (the LOD was NOT determined using inverse calibration)
y_true = principal_Df.loc[:, 'principal component 1']
y_pred = list(map(myfunc, x))
rmse = mean_squared_error(y_true, y_pred, squared=False)

#calculation of cLOD including also the leverage and RMSE

cLOD = (kLOD*rmse*math.sqrt(1+leverage))/m
print("cLOD for PC1 vs. concentration plot with RMSE and leverage: ",cLOD)

#calculation of cLOD including also the leverage and stdblank

cLOD = (kLOD*stdblank*math.sqrt(1+leverage))/m
print("cLOD for PC1 vs. concentration plot with STDblank and leverage: ",cLOD)
```

```
yLOD for PC1 vs. concentration plot:  -0.3215183587656165
                     PC1  Concentration
Concentration                          
0.0            -7.344232            0.0
500.0          -5.570894          500.0
1000.0         -5.404290         1000.0
2000.0         -5.150209         2000.0
4000.0         -0.712469         4000.0
6000.0          3.107914         6000.0
9000.0         10.798600         9000.0
12000.0         5.671004        12000.0
15000.0        13.394979        15000.0
```

```
cLOD for PC1 vs. concentration plot with RMSE and leverage:  9984.204246604699
cLOD for PC1 vs. concentration plot with STDblank and leverage:  5178.195007887557
```

In [23]:

```
############################## Methods based on PLSR and PCR ##############################
```

In [24]:

```
#PCR

from sklearn.model_selection import StratifiedKFold
from sklearn.linear_model import LinearRegression
import math
import statistics

#list for number of components that yielded to the minimum R2/RMSE
numcomp = []
#copy of the feature DataFrame
ff = features.copy()
#to apply stratified k-fold method from sklearn
ff.iloc[:,-1] = ff.iloc[:,-1].astype("string")

#repeat the stratified k-fold validation 10 times with shuffled data
for c in range(0,11):
    ff = ff.sample(frac=1).reset_index(drop=True)
    X = ff.iloc[:,:-1]
    y = ff.iloc[:,-1]

    loo = StratifiedKFold(n_splits=5)

    loo.get_n_splits(X,y)

    r2list = []
    rmselist = []

    #repeat stratified k-fold for different number of components
    for n in range(1,20):

        pca = PCA(n_components=n)
        regr = LinearRegression()
        r2mean = 0
        rmsemean = 0

        for i, (train_index, test_index) in enumerate(loo.split(X,y)):

            #first normalize the features of the training set
            norm_features = X.iloc[train_index,:].copy()
            mean = norm_features.iloc[:,:].mean()
            std = norm_features.iloc[:,:].std()
            norm_features.iloc[:,:] = (norm_features.iloc[:,:]-mean)/std
            norm_features_test = X.iloc[test_index,:].copy()
            norm_features_test.iloc[:,:] = (norm_features_test.iloc[:,:]-mean)/std

            #first fit on training data and the transform test data
            principalComponentstrain = pca.fit_transform(norm_features.iloc[:,:])
            principalComponentstest = pca.transform(norm_features_test.iloc[:,:])

            #build regression model on PCA components
            reg = regr.fit(principalComponentstrain, y.iloc[train_index])

            #calculate R2 and rmse
            r2 = reg.score(principalComponentstest, y.iloc[test_index]) #Q2
            y_pred = reg.predict(principalComponentstest)
            rmse = mean_squared_error(y.iloc[test_index], y_pred, squared=False) #squared=False --> RMSE
            r2mean += r2
            rmsemean += rmse
        r2mean=(r2mean/5)
        rmsemean=(rmsemean/5)
        r2list.append(r2mean)
        rmselist.append(rmsemean)

    xs = [x for x in range(1,len(r2list)+1)]

    #plot the R2 for the different number of components
    plt.plot(xs, r2list)
    plt.ylabel('Q2 (i.e., R2 Prediction)')
    plt.xlabel('Number of Components')
    plt.show()
    plt.close()

    #Minimum number of components for smallest RMSE
    comp1 = rmselist.index(min(rmselist))+1
    print("Minimum number of components for smallest RMSE:", comp1, "(For RMSE of:", min(rmselist), ")")
    numcomp.append(comp1)

    #Minimum number of components for greatest Q2
    comp2 = r2list.index(max(r2list))+1
    print("Minimum number of components for greatest Q2:", comp2, "(For Q2 of:", max(r2list), ")")
    numcomp.append(comp2)

#Calculation of LOD
pcr = LinearRegression()
#use the number of components previously determined
pca = PCA(n_components=round(statistics.median(numcomp)))
print("Number of components:",round(statistics.median(numcomp)))
components = pca.fit_transform(normalized_features.iloc[:,:-1])

pcr.fit(components, normalized_features.iloc[:,-1])

#get R2 and RMSE of the fitted model
r2 = pcr.score(components, normalized_features.iloc[:,-1])
y_pred = pcr.predict(components)
rmse = mean_squared_error(normalized_features.iloc[:,-1], y_pred, squared=False)
print("R2:",r2,"RMSE:",rmse)

plt.figure(figsize=(5,5))
predicted = pcr.predict(components)
actual = normalized_features.iloc[:,-1]
plt.scatter(actual, predicted, c='crimson', s=3)
plt.xlabel('True Values (Concentration [ppm])')#, fontsize=15)
plt.ylabel('Predictions (Concentration [ppm])')#, fontsize=15)
plt.axis('equal')
plt.show()
plt.close()

#calculation of cLOD including also the leverage and STDblank/RMSE of predicted vs actual
rmse = mean_squared_error(actual, predicted, squared=False)

#use the slope of the "predicted=actual"-line
m = 1

cLOD = (kLOD*rmse*math.sqrt(1+leverage))/m
print("cLOD for PCR with leverage and RMSE:",cLOD)

#calculation of cLOD including also the leverage and stdblank of predicted vs actual
stdblank = predicted[actual[actual == 0].index].std()

cLOD = (kLOD*stdblank*math.sqrt(1+leverage))/m
print("cLOD for PCR with leverage and STDblank:",cLOD)
```

```
Minimum number of components for smallest RMSE: 17 (For RMSE of: 1017.254524824771 )
Minimum number of components for greatest Q2: 17 (For Q2 of: 0.9556899884007674 )
```

```
Minimum number of components for smallest RMSE: 15 (For RMSE of: 972.1091050285643 )
Minimum number of components for greatest Q2: 15 (For Q2 of: 0.9588500013620938 )
```

```
Minimum number of components for smallest RMSE: 19 (For RMSE of: 966.9406524559408 )
Minimum number of components for greatest Q2: 19 (For Q2 of: 0.9575595311532688 )
```

```
Minimum number of components for smallest RMSE: 14 (For RMSE of: 1013.5557494951447 )
Minimum number of components for greatest Q2: 14 (For Q2 of: 0.9547137188950258 )
```

```
Minimum number of components for smallest RMSE: 19 (For RMSE of: 995.2864201773116 )
Minimum number of components for greatest Q2: 19 (For Q2 of: 0.9574235322599588 )
```

```
Minimum number of components for smallest RMSE: 15 (For RMSE of: 1011.2227035995593 )
Minimum number of components for greatest Q2: 15 (For Q2 of: 0.956084344839805 )
```

```
Minimum number of components for smallest RMSE: 15 (For RMSE of: 1030.752163771012 )
Minimum number of components for greatest Q2: 15 (For Q2 of: 0.9531868578506302 )
```

```
Minimum number of components for smallest RMSE: 16 (For RMSE of: 1103.722804352346 )
Minimum number of components for greatest Q2: 14 (For Q2 of: 0.9472624207090492 )
```

```
Minimum number of components for smallest RMSE: 15 (For RMSE of: 1018.8097132877522 )
Minimum number of components for greatest Q2: 15 (For Q2 of: 0.9534738247765512 )
```

```
Minimum number of components for smallest RMSE: 14 (For RMSE of: 1033.5580980405541 )
Minimum number of components for greatest Q2: 14 (For Q2 of: 0.9531480000378642 )
```

```
Minimum number of components for smallest RMSE: 15 (For RMSE of: 1054.000648600602 )
Minimum number of components for greatest Q2: 16 (For Q2 of: 0.9507723172437238 )
Number of components: 15
R2: 0.9693813051710966 RMSE: 849.3829729443188
```

```
cLOD for PCR with leverage and RMSE: 3189.065499286704
cLOD for PCR with leverage and STDblank: 2935.4457325117837
```

In [25]:

```
#PLSR

from sklearn.model_selection import StratifiedKFold
from sklearn.cross_decomposition import PLSRegression

numcomp = []
ff = features.copy()
#Change datatype to string to apply StratifiedKFold-Method
ff.iloc[:,-1] = ff.iloc[:,-1].astype("string")

for c in range(0,11):
    ff = ff.sample(frac=1).reset_index(drop=True)
    X = ff.iloc[:,:-1]
    y = ff.iloc[:,-1]

    loo = StratifiedKFold(n_splits=5)
    loo.get_n_splits(X,y)

    r2list = []
    rmselist = []

    for n in range(1,20):

        pls33 = PLSRegression(n_components=n)
        r2mean = 0
        rmsemean = 0

        for i, (train_index, test_index) in enumerate(loo.split(X,y)):

            norm_features = X.iloc[train_index,:].copy()
            mean = norm_features.iloc[:,:].mean()
            std = norm_features.iloc[:,:].std()
            norm_features.iloc[:,:] = (norm_features.iloc[:,:]-mean)/std
            norm_features_test = X.iloc[test_index,:].copy()
            norm_features_test.iloc[:,:] = (norm_features_test.iloc[:,:]-mean)/std
            
            pls33.fit(norm_features.iloc[:,:], y.iloc[train_index])
            r2 = pls33.score(norm_features_test.iloc[:,:], y.iloc[test_index]) #Q2
            y_pred = pls33.predict(norm_features_test.iloc[:,:])
            rmse = mean_squared_error(y.iloc[test_index], y_pred, squared=False) #squared=False --> RMSE
            r2mean += r2 
            rmsemean += rmse
        r2mean=(r2mean/5)
        rmsemean=(rmsemean/5)
        r2list.append(r2mean)
        rmselist.append(rmsemean)

    xs = [x for x in range(1,len(r2list)+1)]

    plt.plot(xs, rmselist)
    plt.ylabel('RMSE')
    plt.xlabel('Number of Components')
    plt.show()
    plt.close()

    #Minimum number of components for smallest RMSE
    comp1 = rmselist.index(min(rmselist))+1
    print("Minimum number of components for smallest RMSE:", comp1, "(For RMSE of:", min(rmselist), ")")
    numcomp.append(comp1)

    #Minimum number of components for greatest Q2
    comp2 = r2list.index(max(r2list))+1
    print("Minimum number of components for greatest Q2:", comp2, "(For Q2 of:", max(r2list), ")")
    numcomp.append(comp2)

#Now calculation of LOD
plsr = PLSRegression(n_components=round(statistics.median(numcomp)))
print("Number of components:",round(statistics.median(numcomp)))
plsr.fit(normalized_features.iloc[:,:-1], normalized_features.iloc[:,-1])

r2 = plsr.score(normalized_features.iloc[:,:-1], normalized_features.iloc[:,-1])
y_pred = plsr.predict(normalized_features.iloc[:,:-1])
rmse = mean_squared_error(normalized_features.iloc[:,-1], y_pred, squared=False)
print("R2:",r2,"RMSE:",rmse)

plt.figure(figsize=(5,5))
predicted = plsr.predict(normalized_features.iloc[:,:-1])
actual = normalized_features.iloc[:,-1]
plt.scatter(actual, predicted, c='crimson', s=3)
plt.xlabel('True Values (Concentration [ppm])')
plt.ylabel('Predictions (Concentration [ppm])')
plt.axis('equal')
plt.show()
plt.close()

#calculation of cLOD including also the leverage and RMSE of predicted vs actual

rmse = mean_squared_error(actual, predicted, squared=False)

m = 1

cLOD = (kLOD*rmse*math.sqrt(1+leverage))/m
print("cLOD for PLSR with leverage and RMSE:",cLOD)

#calculation of cLOD including also the leverage and stdblank of predicted vs actual
stdblank = predicted[actual[actual == 0].index].std()

cLOD = (kLOD*stdblank*math.sqrt(1+leverage))/m
print("cLOD for PLSR with leverage and STDblank:",cLOD)
```

```
Minimum number of components for smallest RMSE: 9 (For RMSE of: 978.3016137398643 )
Minimum number of components for greatest Q2: 9 (For Q2 of: 0.9590800257507379 )
```

```
Minimum number of components for smallest RMSE: 9 (For RMSE of: 894.5610534164946 )
Minimum number of components for greatest Q2: 9 (For Q2 of: 0.9657846529758096 )
```

```
Minimum number of components for smallest RMSE: 9 (For RMSE of: 903.1105820856301 )
Minimum number of components for greatest Q2: 9 (For Q2 of: 0.9645957545790521 )
```

```
Minimum number of components for smallest RMSE: 8 (For RMSE of: 1028.5024590805592 )
Minimum number of components for greatest Q2: 8 (For Q2 of: 0.9532923160423854 )
```

```
Minimum number of components for smallest RMSE: 10 (For RMSE of: 899.6413151996783 )
Minimum number of components for greatest Q2: 10 (For Q2 of: 0.9637563551334839 )
```

```
Minimum number of components for smallest RMSE: 8 (For RMSE of: 1009.975521904152 )
Minimum number of components for greatest Q2: 8 (For Q2 of: 0.9550832223781317 )
```

```
Minimum number of components for smallest RMSE: 8 (For RMSE of: 967.8999135391465 )
Minimum number of components for greatest Q2: 8 (For Q2 of: 0.9588651410874952 )
```

```
Minimum number of components for smallest RMSE: 10 (For RMSE of: 923.489717040013 )
Minimum number of components for greatest Q2: 10 (For Q2 of: 0.9632934487487855 )
```

```
Minimum number of components for smallest RMSE: 8 (For RMSE of: 979.6255059138417 )
Minimum number of components for greatest Q2: 8 (For Q2 of: 0.9586915915404639 )
```

```
Minimum number of components for smallest RMSE: 8 (For RMSE of: 954.9681448986385 )
Minimum number of components for greatest Q2: 8 (For Q2 of: 0.9601352812556208 )
```

```
Minimum number of components for smallest RMSE: 8 (For RMSE of: 1016.0758363771969 )
Minimum number of components for greatest Q2: 8 (For Q2 of: 0.9547620532225147 )
Number of components: 8
R2: 0.9770151634401849 RMSE: 735.9205383361036
```

```
cLOD for PLSR with leverage and RMSE: 2763.063157351539
cLOD for PLSR with leverage and STDblank: 2697.102280499917
```

In [ ]:

```

```
