# Supplementary material for: Methods for Estimating the Detection and Quantification Limits of Key Substances in Beer Maturation with Electronic Noses
Source: Sensors (Basel). 2024 May 30;24(11):3520. doi: 10.3390/s24113520 (PMC11175341; doi:10.3390/s24113520)
Supplement: Supplementary file 1 [file sensors-24-03520-s001.zip › Supplementary Figures and Tables.pdf]

Supplementary Figures and Tables

Methods for Estimating the Detection and Quantification Limits of Key Substances in Beer Maturation with Electronic Noses

Experimental Setup

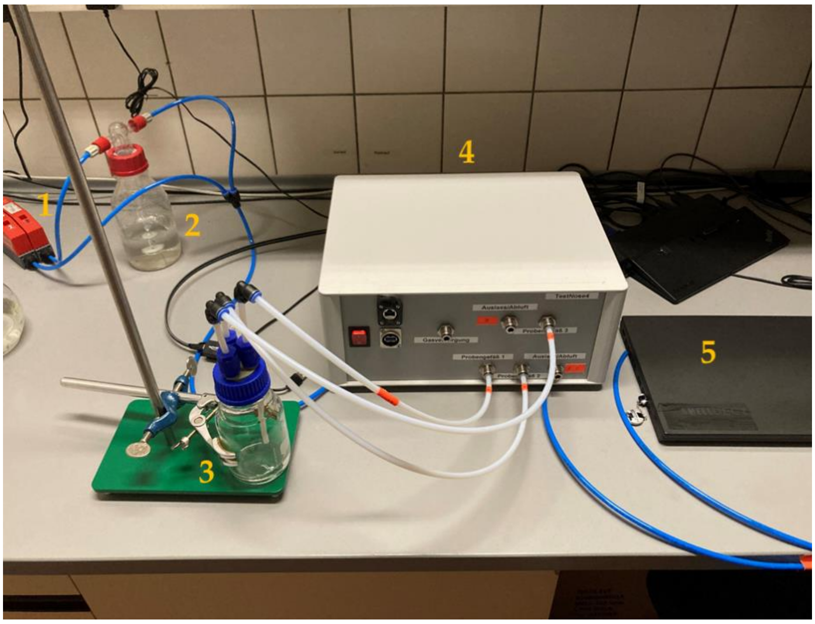

Figure S1: The mass flow controllers (1) allow to mix dry air and humid air (2) for controlling the humidity of the carrier gas directed to the electronic nose (4). The sample vessel (3) is connected to the electronic nose by three tubes. These are used to remove ambient air in the vessel and to direct sample air to the sensor. The notebook (5) was used to control the system and read out the data.

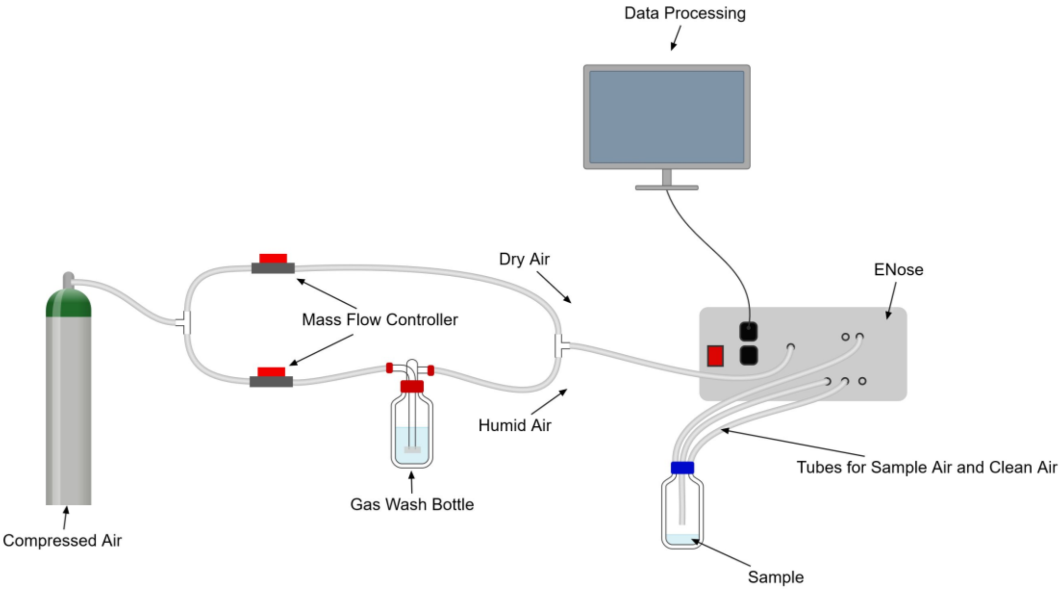

Figure S2: Compressed air from a gas cylinder was used as clean air as carrier gas and for sensor recovery. Mass flow controllers were used to control humidity by mixing dry and humid air. The electronic nose is connected by three tubes with the sample: one tube allows the clean air to be fed into the sample vessel; A second tube is used to direct the sample air to the sensors; the third tube directs the air out of the device without washing over the sensors - this allows ambient air to be removed from the sample vessel.

Diacetyl

Table S1: Number of measurements conducted for each concentration level of diacetyl. The concentration of 0.00 ppm comprises the measurements of the 5% v/v ethanol-water solution.

Concentration [ppm]	No. of measurements
0.00	6
0.05	6
0.10	6
0.20	6
0.50	6
1.00	6

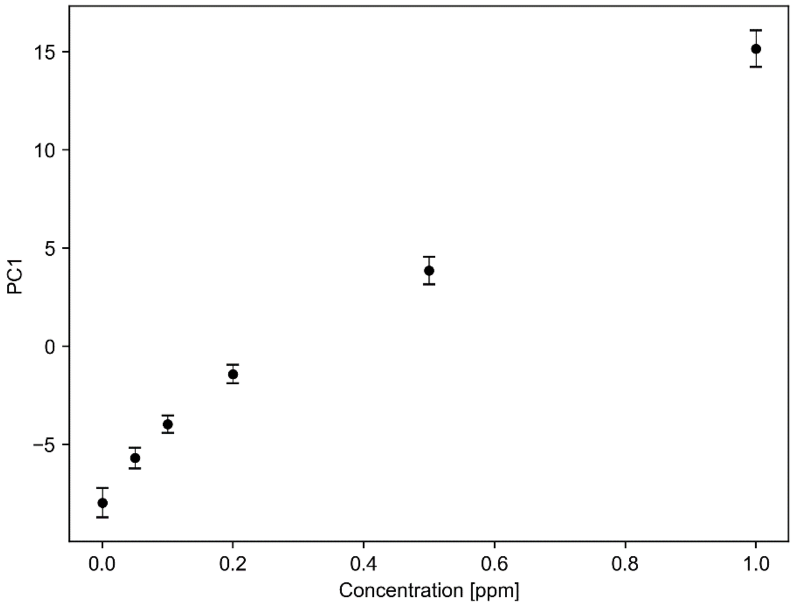

Figure S3: The first principal component (PC1) plotted over the concentration levels of diacetyl. PC1 explained 99.59% of the variance of the data. The error bars correspond to the mean and standard deviation of the measurements per concentration level.

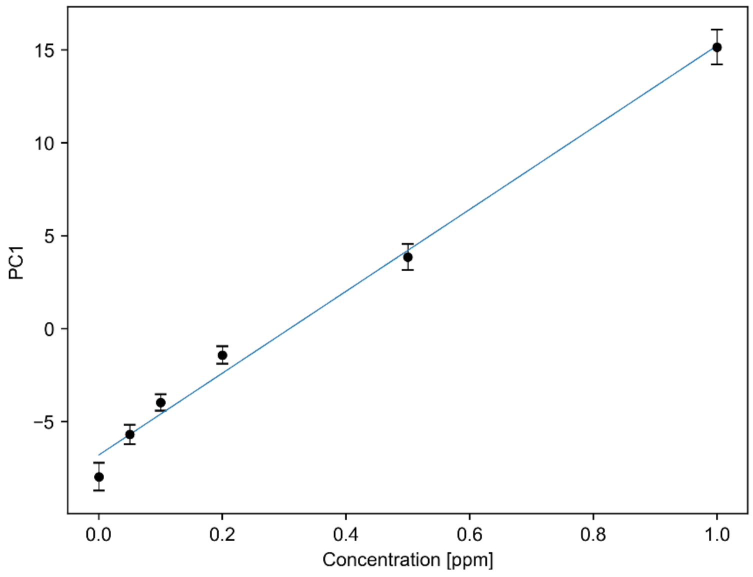

Figure S4: The first principal component (PC1) plotted over the concentration levels of diacetyl. PC1 explained 99.59% of the variance of the data. The error bars correspond to the mean and standard deviation of the measurements per concentration level. The calibration curve was calculated using OLS.

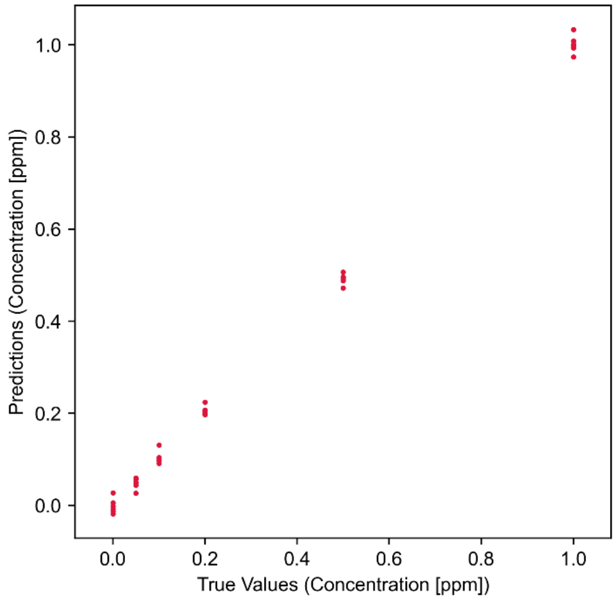

Figure S5: Predicted and true values of the concentration of diacetyl for the PCR model with 10 components and a Q^2 of 0.9973 and an RMSE of 0.018.

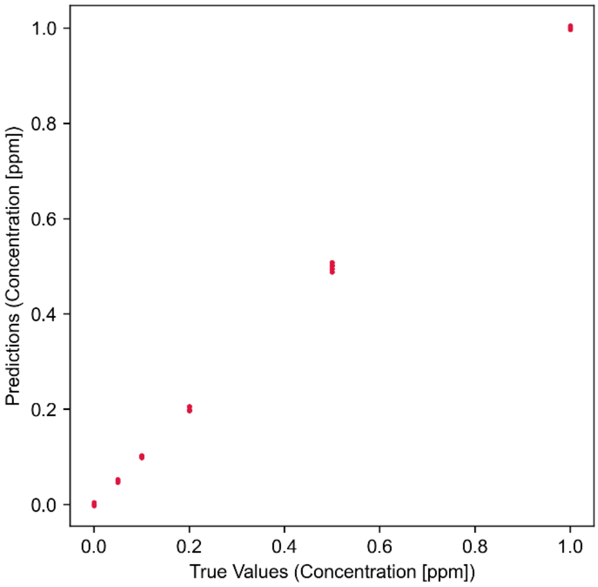

Figure S6: Predicted and true values of the concentration of diacetyl for the PLSR model with 11 components and a Q^2 of 0.9999 and an RMSE of 0.004.

Ethyl acetate

Table S2: Number of measurements conducted for each concentration level of ethyl acetate. The concentration of 0 ppm comprises the measurements of the 5% v/v ethanol-water solution.

**Concentration [ppm]**	**No. of measurements**
0	15
500	8
1,250	9
2,500	9
5,000	9
7,500	9
10,000	8

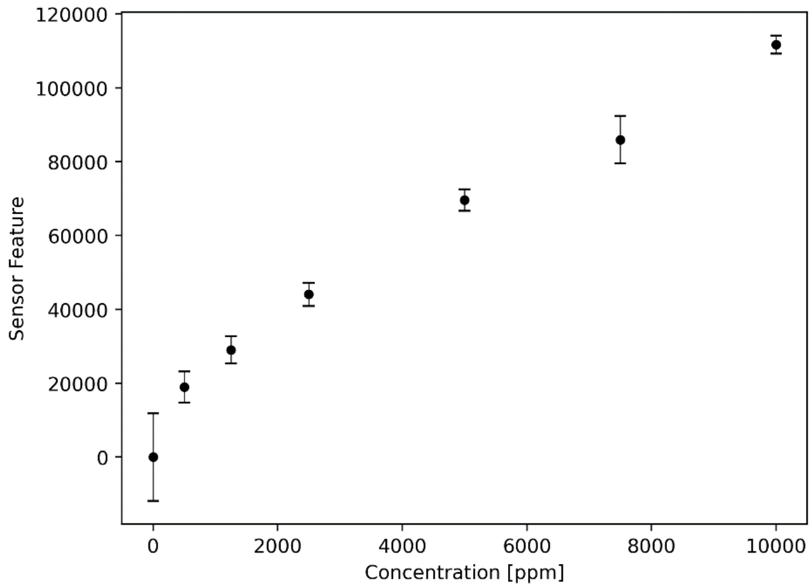

Figure S7: Exemplary feature values for sensor 1 over the concentration levels of ethyl acetate for a single measurement day. The error bars correspond to the mean and standard deviation of the measurements per concentration level.

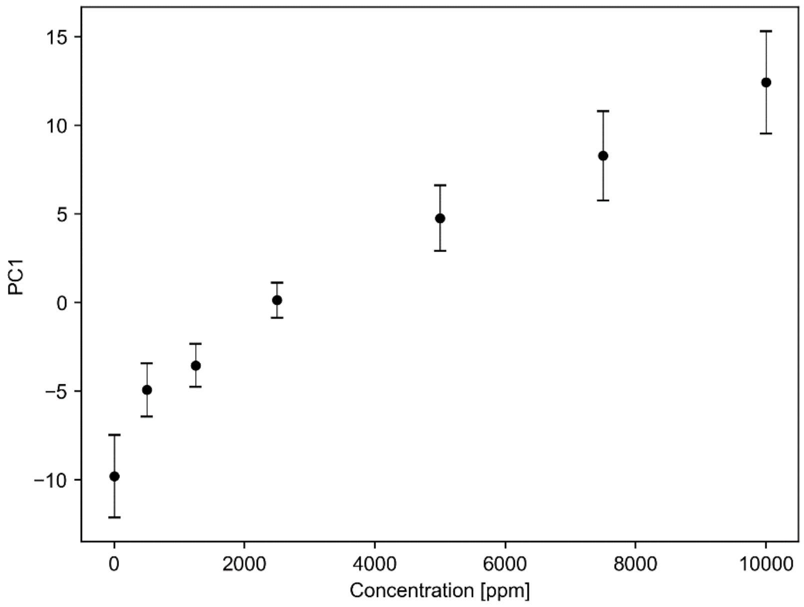

Figure S8: The first principal component (PC1) plotted over the concentration levels of ethyl acetate. PC1 explained 98.95% of the variance of the data. The error bars correspond to the mean and standard deviation of the measurements per concentration level.

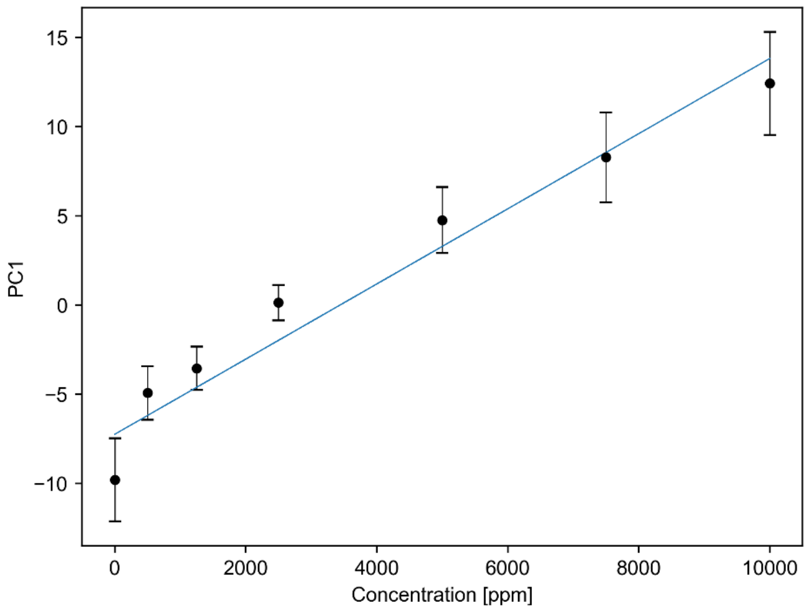

Figure S9: The first principal component (PC1) plotted over the concentration levels of ethyl acetate. PC1 explained 98.95% of the variance of the data. The error bars correspond to the mean and standard deviation of the measurements per concentration level. The calibration curve was calculated using OLS.

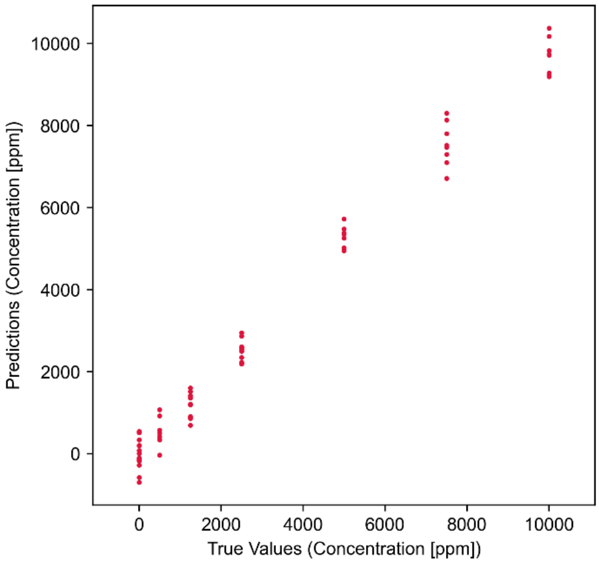

Figure S10: Predicted and true values of the concentration of ethyl acetate for the PCR model with 19 components and a Q^2 of 0.9886 and an RMSE of 371.897.

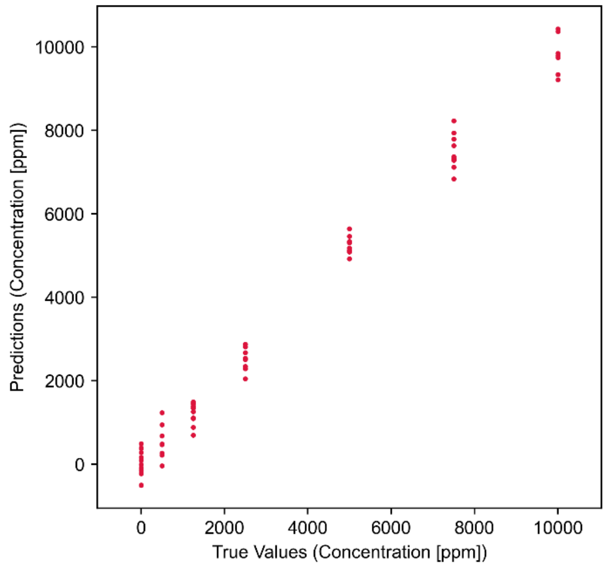

Figure S11: Predicted and true values of the concentration of ethyl acetate for the PLSR model with 7 components and a Q^2 of 0.9899 and an RMSE of 349.168.

2-Phenylethanol

Table S3: Number of measurements conducted for each concentration level of 2-Phenylethanol. The concentration of 0 ppm comprises the measurements of the 5% v/v ethanol-water solution.

**Concentration [ppm]**	**No. of measurements**
0	9
10	9
50	9
100	9
150	9
200	9

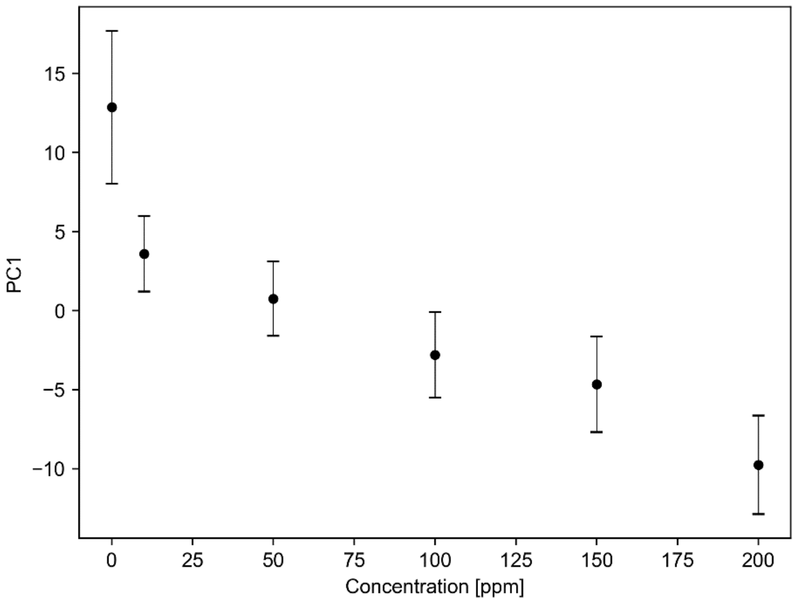

Figure S12: The first principal component (PC1) plotted over the concentration levels of 2-Phenylethanol. PC1 explained 97.91% of the variance of the data. The error bars correspond to the mean and standard deviation of the measurements per concentration level.

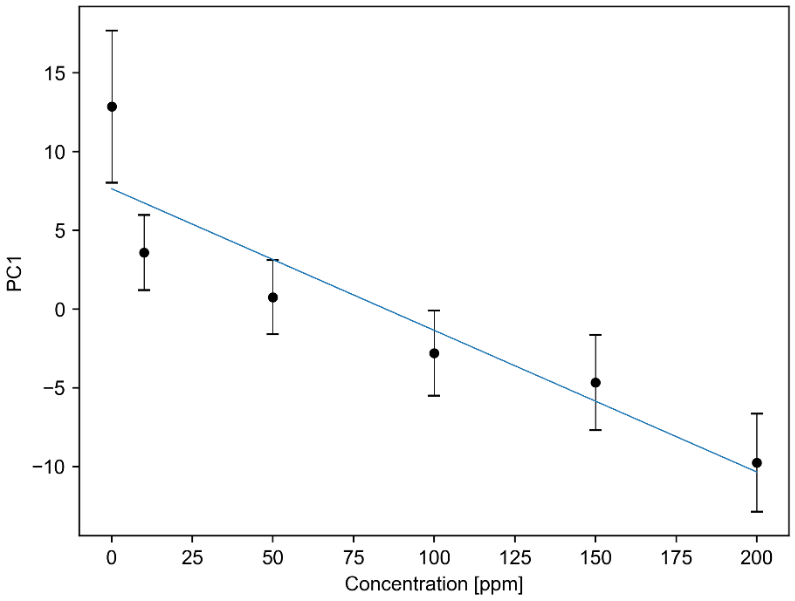

Figure S13: The first principal component (PC1) plotted over the concentration levels of 2-Phenylethanol. PC1 explained 98.95% of the variance of the data. The error bars correspond to the mean and standard deviation of the measurements per concentration level. The calibration curve was calculated using OLS.

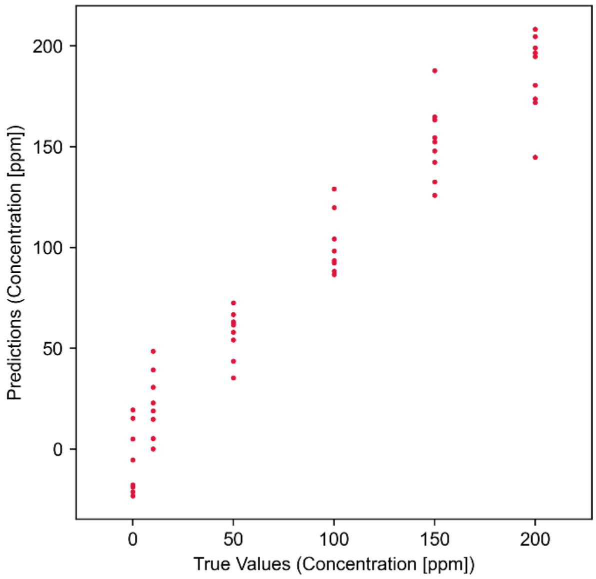

Figure S14: Predicted and true values of the concentration of 2-Phenylethanol for the PCR model with 19 components and a Q^2 of 0.9406 and an RMSE of 17.729.

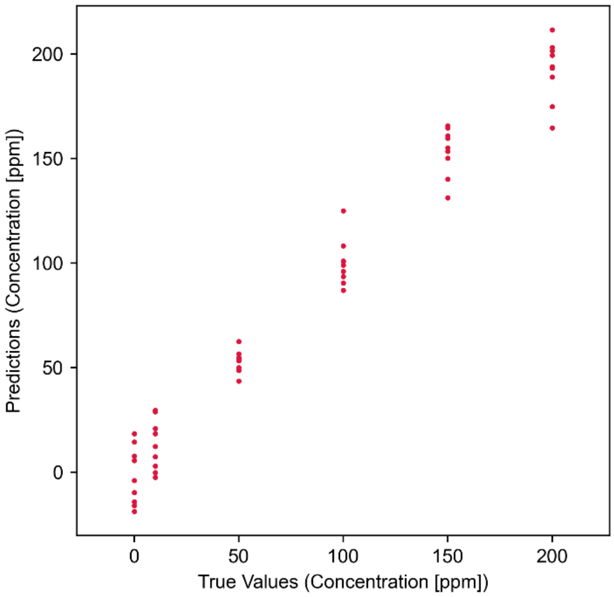

Figure S15: Predicted and true values of the concentration of 2-Phenylethanol for the PLSR model with 8 components and a Q^2 of 0.9736 and an RMSE of 11.819.

Isobutanol

Table S4: Number of measurements conducted for each concentration level of isobutanol. The concentration of 0 ppm comprises the measurements of the 5% v/v ethanol-water solution.

**Concentration [ppm]**	**No. of measurements**
0	10
250	9

500	9
1,000	9
2,000	9

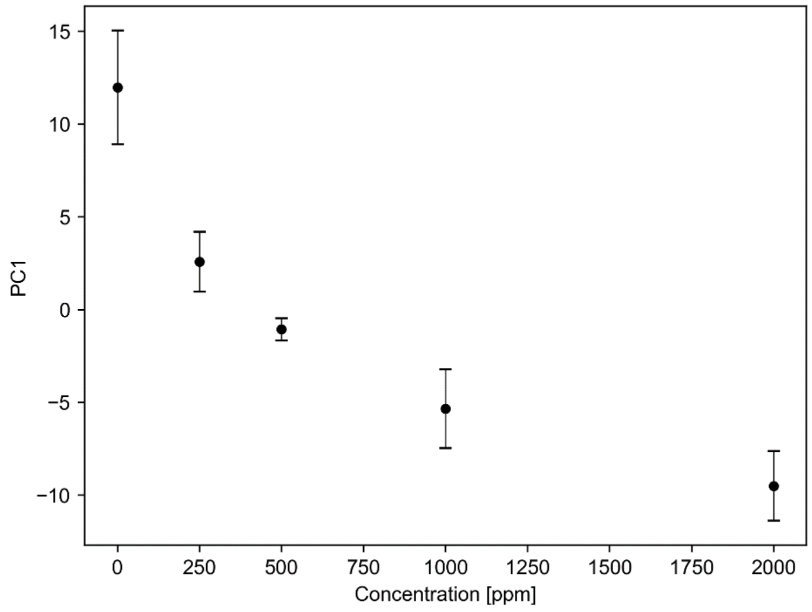

Figure S16: The first principal component (PC1) plotted over the concentration levels of isobutanol. PC1 explained 98.44% of the variance of the data. The error bars correspond to the mean and standard deviation of the measurements per concentration level.

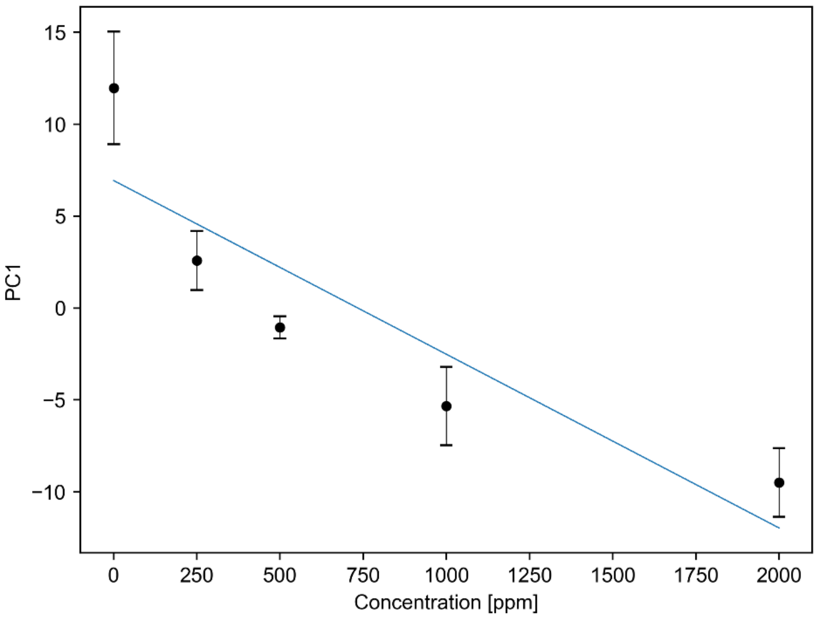

Figure S17: The first principal component (PC1) plotted over the concentration levels of isobutanol. PC1 explained 98.44% of the variance of the data. The error bars correspond to the mean and standard deviation of the measurements per concentration level. The calibration curve was calculated using OLS.

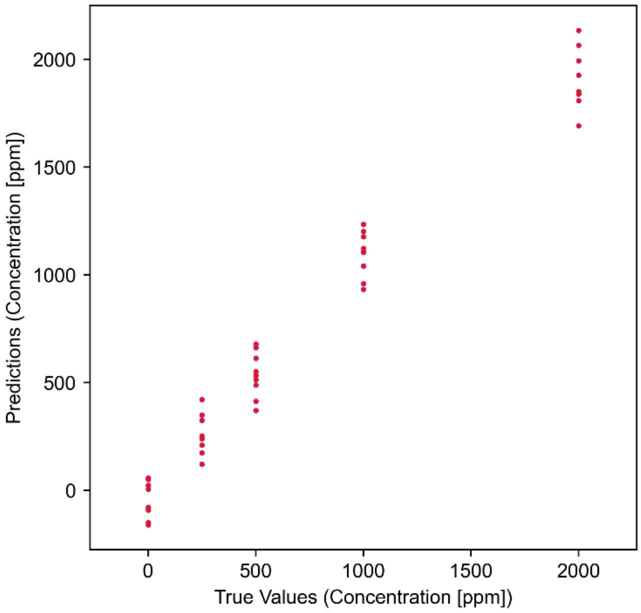

Figure S18: Predicted and true values of the concentration of isobutanol for the PCR model with 15 components and a Q^2 of 0.9717 and an RMSE of 119.021.

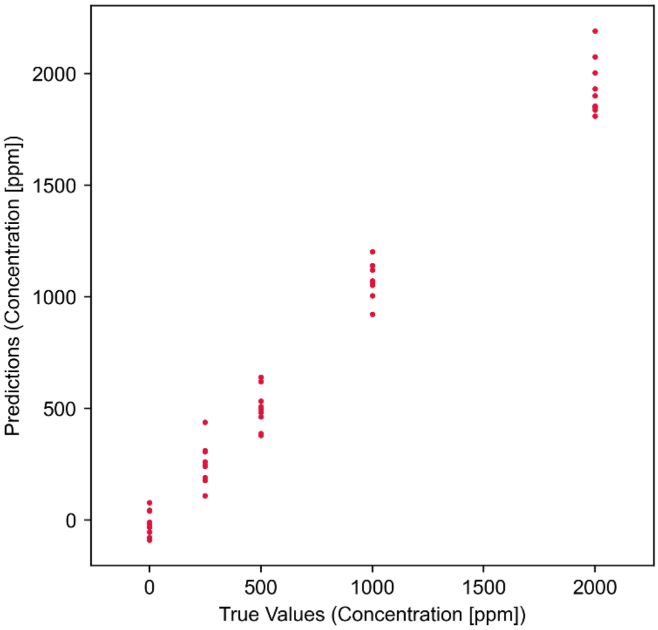

Figure 19: Predicted and true values of the concentration of isobutanol for the PLSR model with 7 components and a Q^2 of 0.9815 and an RMSE of 96.189.

Acetaldehyde

Table S5: Number of measurements conducted for each concentration level of acetaldehyde. The concentration of 0 ppm comprises the measurements of the 5% v/v ethanol-water solution.

**Concentration [ppm]**	**No. of measurements**
0	16
500	13
1,000	12
2,000	11
4,000	12
6,000	13
9,000	10
12,000	9
15,000	9

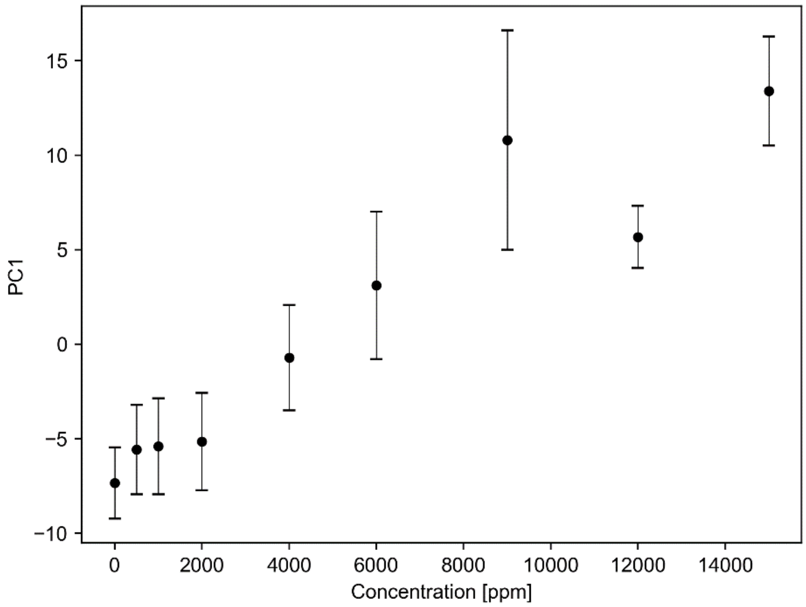

Figure S20: The first principal component (PC1) plotted over the concentration levels of acetaldehyde. PC1 explained 93.47% of the variance of the data. The error bars correspond to the mean and standard deviation of the measurements per concentration level. A high standard deviation of the measurements can be observed.

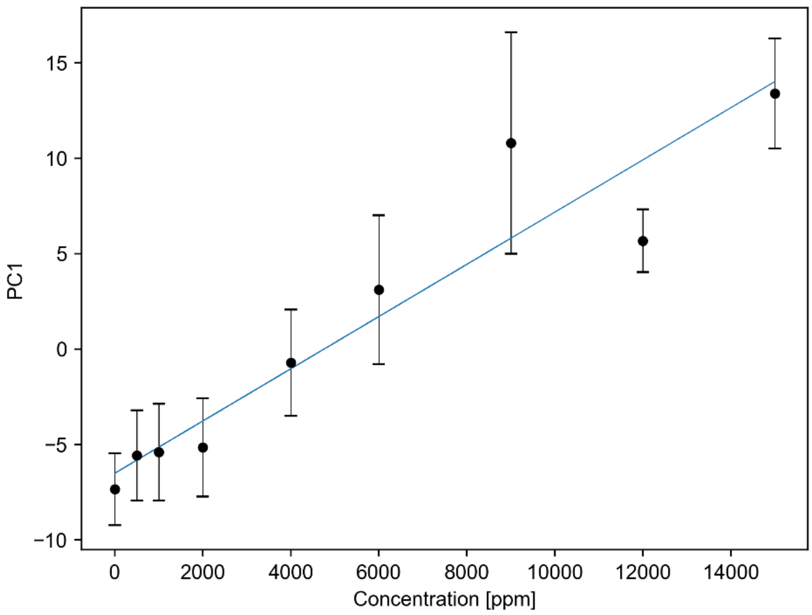

Figure S21: The first principal component (PC1) plotted over the concentration levels of acetaldehyde. PC1 explained 93.47% of the variance of the data. The error bars correspond to the mean and standard deviation of the measurements per concentration level. The calibration curve was calculated using OLS.

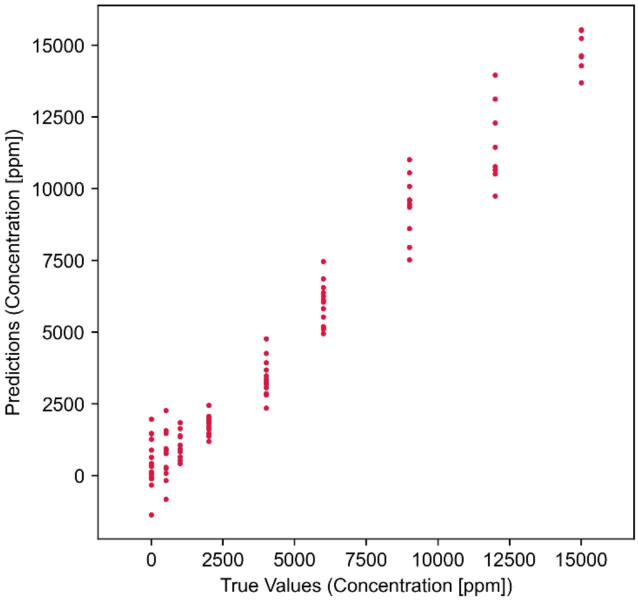

Figure S22: Predicted and true values of the concentration of acetaldehyd for the PCR model with 16 components and a Q2 of 0.9694 and an RMSE of 848.776.

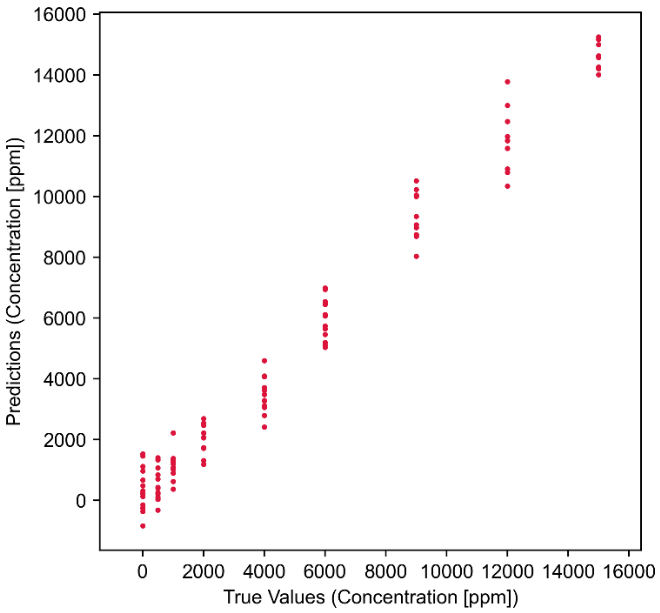

Figure S23: Predicted and true values of the concentration of acetaldehyd for the PCR model with 9 components and a Q2 of 0.9799 and an RMSE of 688.082.
